# Pro-inflammatory microenvironment and systemic accumulation of CXCR3+ cell exacerbate lung pathology of old rhesus macaques infected with SARS-CoV-2

**DOI:** 10.1038/s41392-021-00734-w

**Published:** 2021-09-01

**Authors:** Hong-Yi Zheng, Xiao-Yan He, Wei Li, Tian-Zhang Song, Jian-Bao Han, Xiang Yang, Feng-Liang Liu, Rong-Hua Luo, Ren-Rong Tian, Xiao-Li Feng, Yu-Hua Ma, Chao Liu, Ming-Hua Li, Yong-Tang Zheng

**Affiliations:** 1 Key Laboratory of Animal Models and Human Disease Mechanisms of Chinese Academy of Sciences, KIZ-CUHK Joint Laboratory of Bioresources and Molecular Research in Common Diseases, Kunming Institute of Zoology, Chinese Academy of Sciences, Kunming, Yunnan China; 2 Kunming College of Life Science, University of Chinese Academy of Sciences, Kunming, Yunnan China; 3 Kunming National High-Level Biosafety Research Center for Non-Human Primates, Center for Biosafety Mega-Science, Kunming Institute of Zoology, Chinese Academy of Sciences, Kunming, Yunnan China; 4 National Resource Center for Non-Human Primates, National Research Facility for Phenotypic & Genetic Analysis of Model Animals (Primate Facility), Kunming Institute of Zoology, Chinese Academy of Sciences, Kunming, Yunnan China; 5 Bioland Laboratory (Guangzhou Regenerative Medicine and Health Guangdong Laboratory), Guangzhou, China

**Keywords:** Infectious diseases, Infection

## Abstract

Understanding the pathological features of severe acute respiratory syndrome coronavirus 2 (SARS-CoV-2) infection in an animal model is crucial for the treatment of coronavirus disease 2019 (COVID-19). Here, we compared immunopathological changes in young and old rhesus macaques (RMs) before and after SARS-CoV-2 infection at the tissue level. Quantitative analysis of multiplex immunofluorescence staining images of formalin-fixed paraffin-embedded (FFPE) sections showed that SARS-CoV-2 infection specifically induced elevated levels of apoptosis, autophagy, and nuclear factor kappa-B (NF-κB) activation of angiotensin-converting enzyme 2 (ACE2)+ cells, and increased interferon α (IFN-α)- and interleukin 6 (IL-6)-secreting cells and C-X-C motif chemokine receptor 3 (CXCR3)+ cells in lung tissue of old RMs. This pathological pattern, which may be related to the age-related pro-inflammatory microenvironment in both lungs and spleens, was significantly correlated with the systemic accumulation of CXCR3+ cells in lungs, spleens, and peripheral blood. Furthermore, the ratio of CXCR3+ to T-box protein expression in T cell (T-bet)+ (CXCR3+/T-bet+ ratio) in CD8+ cells may be used as a predictor of severe COVID-19. These findings uncovered the impact of aging on the immunopathology of early SARS-CoV-2 infection and demonstrated the potential application of CXCR3+ cells in predicting severe COVID-19.

## Introduction

Since December 2019, severe acute respiratory syndrome coronavirus 2 (SARS-CoV-2) has dispersed worldwide, causing a variety of clinical syndromes collectively termed coronavirus disease 2019 (COVID-19)^1,2^. The World Health Organization (WHO) declared the disease a pandemic in March 2020. Growing evidence has shown that the pathogenesis of COVID-19 is largely the result of an abnormal host response or overreaction of the immune system. Excessive inflammation can cause serious damage to the lungs and other organs, which primarily manifests as diffuse alveolar damage (DAD) accompanied by platelet-fibrin microthrombi in the pulmonary blood vessels, multiple organ failure, and potential death^3–5^. Acute respiratory distress syndrome (ARDS) is the main cause of death in COVID-19 patients and may be triggered by a cytokine storm^6,7^. Thus, immunopathology plays an important role in disease progression following SARS-CoV-2 infection, and understanding its characteristics is of great significance for the prevention and treatment of COVID-19.

To date, however, most knowledge of COVID-19 pathology comes from autopsy and peripheral blood samples^5,8–11^, and hence the developmental processes of COVID-19 remain poorly understood. Non-human primate (NHP) models provide the opportunity to dynamically study the pathological mechanisms of SARS-CoV-2 infection. Rhesus macaques (RMs, *Macaca mulatta*) are considered a suitable animal model for COVID-19 research as they exhibit similar clinical characteristics to SARS-CoV-2-infected human patients^12–14^. Based on the research of multiple COVID-19 animal models, we identified important pathological features in old RMs compared with young RMs in the previous study, namely delayed but more severe cytokine storm and higher immune cell infiltration^15–17^. We considered that the age-related abnormal immune microenvironment may have been initially formed during early SARS-CoV-2 infection, which subsequently impacted disease progression in old RMs.

In the present study, we evaluated age-related immunopathological changes during SARS-CoV-2 infection from the perspective of viral infection, pathological changes in angiotensin-converting enzyme 2 (ACE2)+ cells, infiltration of inflammatory cells, and functional changes in immune cells. Furthermore, we explored the correlations in immunopathology among lung tissue, spleen tissue, peripheral blood mononuclear cells (PBMCs), and CD8+ cells. Results showed that apoptosis, autophagy, and nuclear factor kappa-B (NF-κB) activation of ACE2+ cells were more serious in old RMs than in young RMs after infection. The increase in interferon (IFN)-α- and interleukin (IL)-6-secreting cells in the lungs and accumulation of C-X-C motif chemokine receptor 3 (CXCR3)+ cells in the lungs, spleens, PBMCs, and CD8+ cells of old RMs may aggravate tissue inflammation. Moreover, owing to the rapid decrease in the frequency of T-box protein expression in T cell (T-bet)+ cells in both PBMCs and CD8+ cells in old RMs after SARS-CoV-2 infection, the CXCR3+/T-bet+ ratio in CD8+ cells exhibits good potential for predicting the severity of COVID-19.

## Results

### SARS-CoV-2-infected RMs exhibited obvious pulmonary infection characteristics

**Fig. 1:**
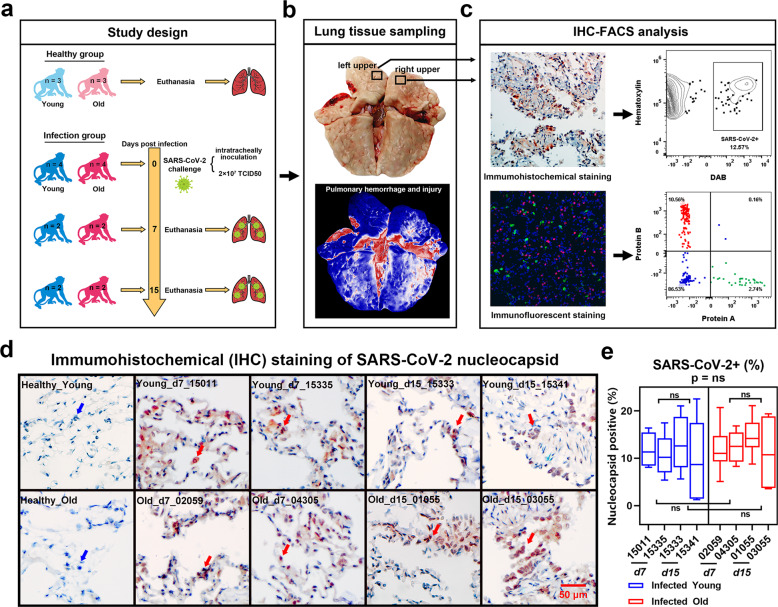
Pathological analysis of lung tissues from SARS-CoV-2-infected young and old RMs. **a** Flow chart showing the grouping of experimental animals and strategy of sample collection. After the intratracheal challenge, four young and four old RMs were killed (half on day 7 and a half on day 15) for lung dissection. Lungs of three healthy young and three healthy old RMs were used as controls. **b** Pseudo-color images (bottom) showing hemorrhage and injury sites (red) and non-obvious lesions (blue) in the lungs. FFPE tissue sections were taken from non-obvious lesions of the upper left and right lungs (top). **c** Microscopy images (left) showing viruses labeled by IHC and proteins labeled by mIF. Two-dimensional scatter diagram (right) showing process of converting microscopy images into flow cytometry data for quantitative analysis using IHC-FACS. **d** Detection of SARS-CoV-2 nucleocapsid antigens by IHC in FFPE sections of healthy and infected RMs. Nuclei indicated by blue arrows are stained dark blue (hematoxylin). Nucleocapsid indicated by the red arrow is stained dark red (DAB). **e** Box plot showing a comparison of SARS-CoV-2+ cell rate in lung tissue sections of young and old RMs. Data calculated by 10 DAB staining images from each animal’s lung tissue sections are shown as median with min to max. *P* value at top of the figure is a comparison of all animals in young and old groups. Square brackets in the figure are comparisons between two groups of animals at different time points. *p* > 0.05 indicates no statistical difference (ns)

We infected four young (5 years old) and four old (16–19 years old) RMs with SARS-CoV-2 by the intratracheal challenge. Half of the animals were euthanized on day 7 and a half on day 15 post infection (DPI) to obtain lung and spleen tissue samples. We also collected corresponding tissue samples from three healthy young (6–11 years old) and three healthy old (25–29 years old) RMs as controls (Fig. 1a). To avoid interference from samples, we only collected tissue from non-obvious lesion areas of the left and right upper lung lobes for the preparation of paraffin sections (Fig. 1b). Subsequently, these sections were subjected to immunohistochemical (IHC) staining of SARS-CoV-2 and multiplex immunofluorescence (mIF) staining of immunological markers. Microscopy images were converted into cell-based digital signal matrixes, which are then analyzed by flow cytometry software (IHC-FACS method) (Fig. 1c).

The IHC staining results revealed virus-infected areas in each lung tissue section of the SARS-CoV-2-infected RMs (Fig. 1d). The frequency of virus-positive cells in the images (200-fold magnification, 627 × 427 μm) of these areas was ~3–22%. Based on analysis of 10 images of each animal, no significant differences in the rate of virus infection were found between the young (11.3%) and old (12.6%) groups or between 7 and 15 DPI (Fig. 1e). Combined with the pathological results of our previous study^15^, both young and old RMs had obvious pulmonary infection characteristics, showing the reliability of our SRAS-CoV-2-infected RM model.

### ACE2+ lung cells in SARS-CoV-2-infected old RMs exhibited severe pathological changes

**Fig. 2:**
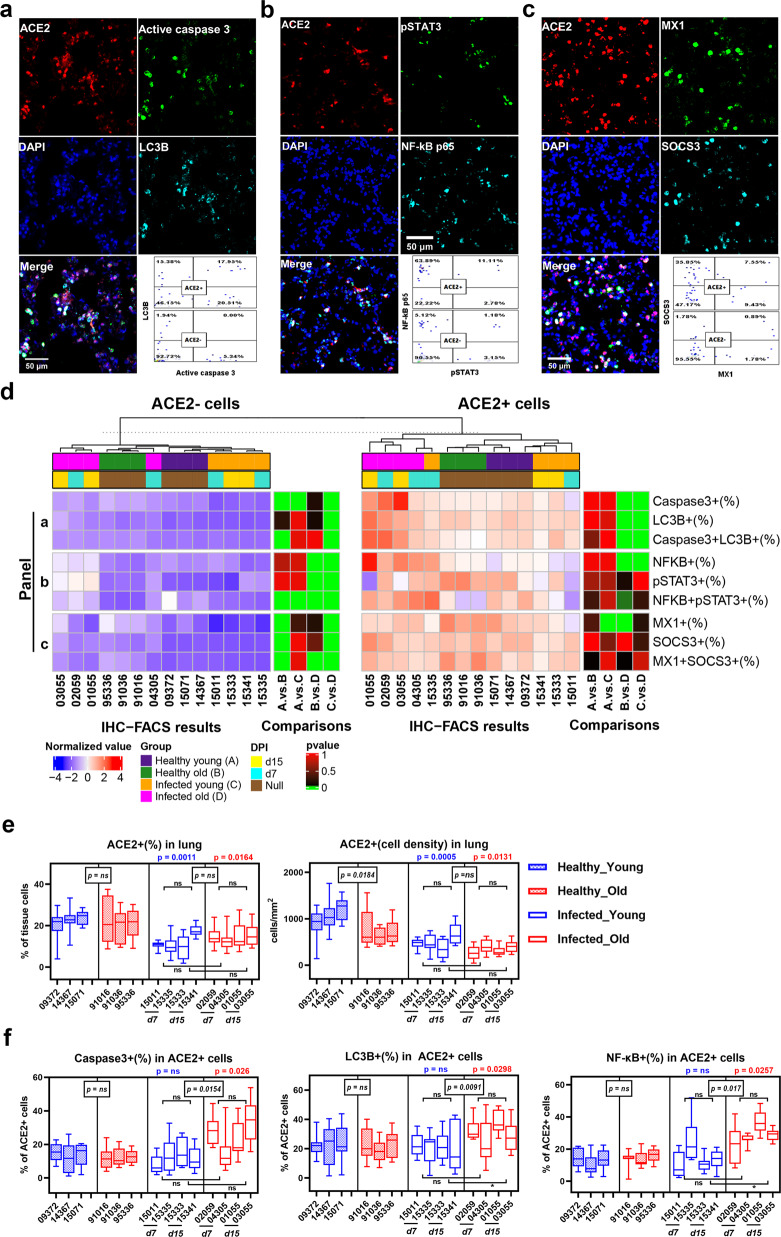
Aggravated pathological changes in ACE2 + cells in lung tissues of old RMs infected with SARS-CoV-2. **a**–**c** mIF images showing three sets of co-staining results of ACE2 with two other proteins in lung tissue sections, representing cell death (**a**), inflammatory signal activation (**b**), and IFN-signal activation (**c**) of ACE2+ and ACE2− cells, respectively. Red, green, and cyan indicate staining of protein markers, and blue DAPI staining indicates nucleus. Two-dimensional scatter diagrams showing the characteristics of ACE2+ and ACE2− cells expressing the other two markers, respectively. **d** Staining results according to **a**, **b**, and **c** of each animal are displayed in heat maps. Cellular components of ACE2+ and ACE2− lung cells are shown in heat maps, respectively: annotation bar indicates group and DPI of each sample; heat map on left represents the normalized average frequency of each cell subsets by R’s scale method, mapped by red-white-blue gradient squares; heat map on right represents *p* values for comparisons between each group (A, healthy young; B, healthy old; C, infected young; D, infected old), and square with grass green was considered to be a statistically significant difference (*p* ≤ 0.05). **e**, **f** Box plots showing frequency and density of ACE2+ lung cells (**e**), and pathological characteristics of ACE2+ (**f**) lung cells in healthy young, healthy old, infected young, and infected old groups. Data calculated using 10 microscopy images of lung tissue sections from each animal are shown as median with min to max. *P* value marked in blue indicates the comparison between infected and healthy young groups, and red indicates the comparison between infected and healthy old groups. Boxed *p* value represents a comparison between young and old RMs in healthy (left) or infected (right) conditions. Square brackets in the figure show a comparison between infected young and old RMs at different time points (7 and 15 DPI). *p ≤ 0.05; ns, *p* > 0.05. Caspase-3, active caspase-3; NF-ΚB, NK-κB p65; pSTAT3, STAT3 (phospho Y705)

Some studies showed that SARS-CoV-2 can affect the apoptosis and autophagy of host cells to promote its own replication, activate the inflammatory signal pathway, such as NF-κB and signal transducer and activator of transcription 3 (STAT3), and induce an abnormal IFN signal, such as boosted expression of MX dynamin-like GTPase (MX1) and suppressor of cytokine signaling 3 (SOCS3), to promote the formation of COVID-19 pathology.^18–22^We next used mIF staining to study the levels of cell death, inflammatory signals, and IFN signals in ACE2+ and ACE2− cells in lung tissue (Fig. 2a–c, Supplementary Fig. 1). First, using IHC-FACS, we found that even in the lung tissue of healthy animals, active cysteinyl aspartate specific proteinase-3 (caspase-3), autophagy-related ubiquitin-like modifier LC3-B (LC3B), NF-κB p65, phospho-STAT3 (pSTAT3), MX1, and SOCS3 were more highly expressed in ACE2+ cells than in ACE2− cells (Fig. 2d, Supplementary Fig. 2). These findings indicate that ACE2+ cells may be a physiologically active cell type. In the healthy groups, no significant differences were found in the frequency of ACE2+ cells between old (21.3%) and young (22.4%) RMs, but the density of ACE2+ cells was significantly lower in old RMs (702.8 cell/mm^2^) than in young RMs (1065 cell/mm^2^; *p* = 0.0184). SARS-CoV-2 infection significantly reduced the frequency and density of ACE2+ cells in both the young and old groups by ~30–50%, but no significant differences were observed between the two groups (young, 12.1%, 495.9 cell/mm^2^, respectively; old, 14.4%, 344.1 cell/mm^2^, respectively) (Fig. 2e).

For ACE2+ lung cells, increased frequencies of caspase-3+ (25.2%), LC3B+ (30.9%), caspase-3+ LC3B+ (13.3%), and NF-κB+ (28.6%) populations were found in the infected old group compared with the healthy control (12.4%, 22.0%, 5.6%, and 14.7%, respectively; *p* < 0.05), but not in the infected young group compared with the healthy young control. Our previous study found that the lung lesions of old RMs were more severe after two weeks of SARS-CoV-2 infection than young RMs^15^. Considering that the frequency of caspase-3+ ACE2+ and NF-κB+ ACE2+ cells in the lungs of old RMs is higher than that in the lungs of young RMs especially at 15 DPI (Fig. 2d, f, Supplementary Fig. 2), and it is likely that the accumulated apoptosis and autophagy of ACE2+ cell aggravates these lesions. In addition, the ACE2− cells in the lungs of old RMs were also impacted by infection and showed high expression of NF-κB (9.6%) and pSTAT3 (11.2%) compared with the healthy control (5.1% and 6.7%, respectively; *p* < 0.05). Furthermore, the frequencies of the MX1+ ACE2− and SOCS3+ ACE2− cells were not affected by infection, but were significantly affected by age, and were highly accumulated in the lung tissues of both healthy and infected old RMs (Fig. 2d, Supplementary Fig. 2). This suggests that the age-related inflammatory microenvironment of lung tissue affects the pathological manifestation of SARS-CoV-2 infection.

### IFN-α- and IL-6-secreting cells markedly increased in old RM lungs after SARS-CoV-2 infection

**Fig. 3:**
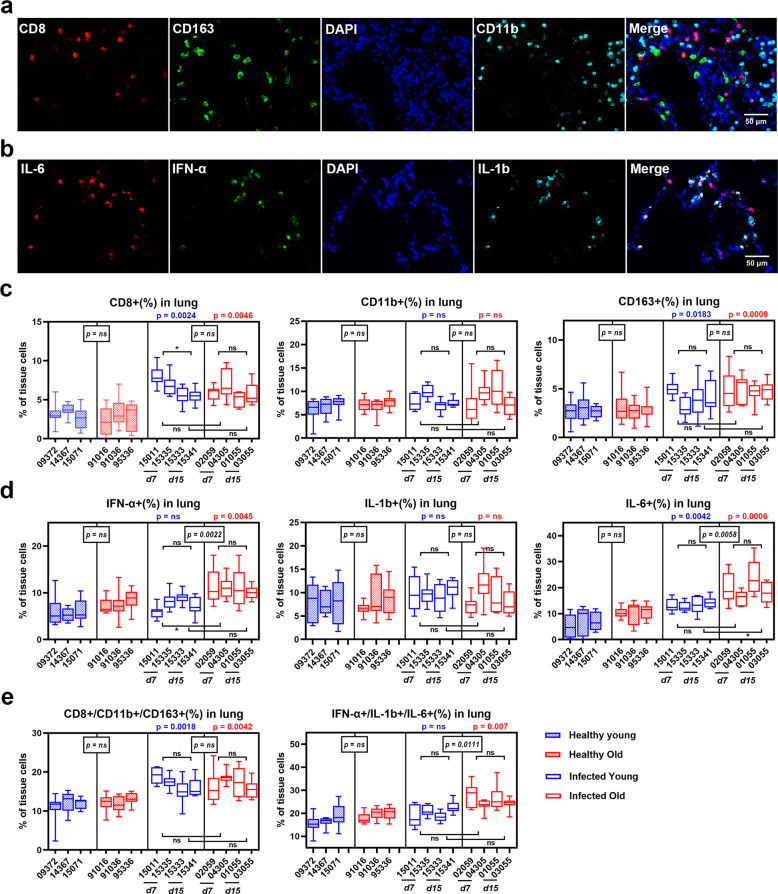
SARS-CoV-2-induced inflammatory cell infiltration in lung tissue of old RMs. **a**, **b** mIF images showing the co-staining results of inflammatory cells (**a**) and inflammatory factors (**b**) in lung tissue sections. Red, green, and cyan indicate staining of protein markers, and blue DAPI staining indicates nucleus. **c**–**e** Box plots showing the staining results according to **a** and **b** in healthy young, healthy old, infected young, and infected old groups. Data calculated using 10 microscopy images of lung tissue sections from each animal are shown as median with min to max. *P* value marked in blue indicates the comparison between infected and healthy young groups, and red indicates the comparison between infected and healthy old groups. Boxed *p* value represents a comparison between young and old RMs in healthy (left) or infected (right) conditions. Square brackets in the figure show a comparison between infected young and old RMs at different time points (7 and 15 DPI). *p ≤ 0.05; ns, *p* > 0.05

In a previous study, we found that the old RMs had a significant increase in inflammatory factors and cells in lung tissues at 2 weeks post SARS-CoV-2 infection^15^. Here, we drive from systemically to locally histological research, further evaluating the inflammatory microenvironment characteristics of lung tissue in animal models. We used mIF staining to mark inflammatory immune cells (CD8, CD163, and CD11b) and inflammatory factor-secreting cells (FN-α, IL-6, and IL-1β) in lung tissue sections (Fig. 3a, b, Supplementary Fig. 3). IHC-FACS analysis showed that the frequency of CD8+ cells and CD163+ macrophages in lung tissue increased dramatically in both young (6.5% and 4.1%, respectively) and old RMs (5.9% and 4.8%, respectively) after infection compared with the healthy young (3.1% and 2.8%, respectively; *p* < 0.05) and old groups (2.9% and 2.8%, respectively; *p* < 0.05). Interestingly, only the infected young group showed a peak in CD8+ cells in lung tissue at 7 DPI (7.4%), followed by a significant decrease at 15 DPI (5.5%; *p* = 0.04) (Fig. 3c, Supplementary Fig. 4). SARS-CoV-2 infection induced an increase in IFN-α+ and IL-6+ cells in the lung tissue of infected old RMs (10.9% and 19.4%, respectively) compared with the healthy old group (7.7% and 10.5%, respectively; *p* < 0.01); however, no significant differences were detected between healthy and infected young RMs. At 7 DPI, the frequency of IFN-α+ cells was significantly higher in the old RMs (11.3%) than in the young RMs (7.1%; *p* = 0.02); at 15 DPI, the frequency of IL-6+ cells was significantly higher in the old RMs (21.0%) than in the young RMs (13.8%; *p* = 0.04) (Fig. 3d, Supplementary Fig. 4). In addition, the aggregation of these cell types demonstrated similar results. Both young and old RMs showed increased infiltration of inflammatory immune cells (CD8+, CD163+, and CD11b+ cells) after infection, whereas inflammatory factor-secreting cells (IFN-α+, IL-6+, and IL-1β+ cells) were only significantly induced in old RMs following infection (Fig. 3e). These results indicate that age affects the infiltration of inflammatory factor-secreting cells in SARS-CoV-2-infected lung tissue and may promote lung lesions in the old RM model.

### SARS-CoV-2 infection induced accumulation of CXCR3+ cells in lung tissues of old RMs

**Fig. 4:**
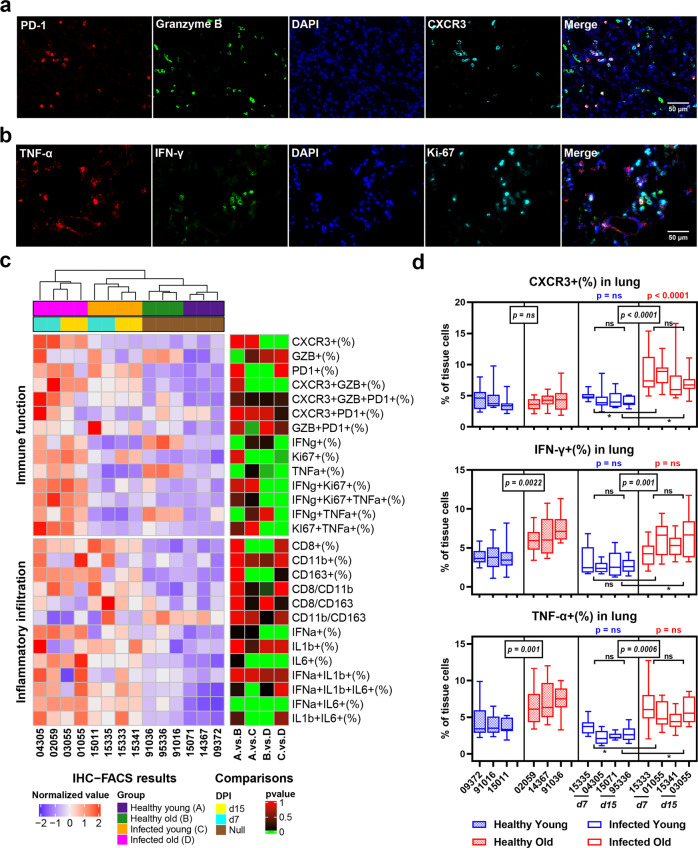
Pro-inflammatory immune microenvironment in lung tissues of old RMs infected with SARS-CoV-2. **a**, **b** mIF images showing the co-staining results of immune function-related proteins in lung tissue sections. Red, green, and cyan indicate staining of protein markers, and blue DAPI staining indicates nucleus. **c** The frequency of inflammatory infiltrating cells and immune function-related cells in the lung tissue of each animal is displayed in a heat map. Annotation bar indicates group and DPI of each sample; heat map on left represents the normalized average frequency of each cell subsets by R’s scale method, mapped by red-white-blue gradient squares; heat map on right represents *p* values for comparisons between each group, and square with grass green was considered to be a statistically significant difference (*p* ≤ 0.05). **d** Box plots showing the frequency of immune function-related cells in healthy young, healthy old, infected young, and infected old groups. Data calculated using 10 microscopy images of lung tissue sections from each animal are shown as median with min to max. *P* value marked in blue indicates the comparison between infected and healthy young groups, and red indicates the comparison between infected and healthy old groups. Boxed *p* value represents the comparison between young and old RMs in healthy (left) or infected (right) conditions. Square brackets in the figure show a comparison between infected young and old RMs at different time points (7 and 15 DPI). *p ≤ 0.05; ns, *p* > 0.05

In addition to changes in the number of inflammatory cells, functional changes in immune cells in the lung inflammatory microenvironment may also be affected by age and SARS-CoV-2 infection. Therefore, we used programmed death-1 (PD-1), granzyme B, and CXCR3 mIF staining to evaluate the regulation, cytotoxicity, and chemotaxis of immune cells, respectively, and tumor necrosis factor-alpha (TNF-α), IFN-γ, and Ki-67 mIF staining to evaluate the pro-inflammatory, antiviral, and proliferation ability of immune cells, respectively (Fig. 4a, b, Supplementary Fig. 3). Results showed that the hierarchical clustering of samples based on the two staining sets examining inflammatory infiltration and immune function perfectly fit their logical groupings, indicating that infection and age strongly impact the immune microenvironment of the lungs. The IHC-FACS results showed that SARS-CoV-2 infection specifically induced an increase in CXCR3+(7.9%) and IFN-γ+ Ki-67+ (2.2%) cells in the lung tissue of old RMs compared with the healthy controls (4.1% and 1.2%, respectively; *p* < 0.0001), but showed no significant differences between the healthy and infected young groups (Fig. 4c, Supplementary Fig. 4). Moreover, CXCR3+ cells were significantly higher in the lung tissue of old RMs at both 7 (8.6%) and 15 DPI (7.2%) than in the lung tissue of young RMs (4.7% and 4.2%, respectively; *p* < 0.0001). These results indicate that rapid and persistent immune cell chemotaxis to the lungs is an important pathological feature of SARS-COV-2 infection in old RMs (Fig. 4d, Supplementary Fig. 4).

Approximately 4% of cells in lung tissue expressed granzyme B, and the frequency was relatively stable, regardless of age and infection. The frequencies of PD-1+ and Ki-67+ cells in the lung tissue were significantly affected by infection but not by age, i.e., were significantly higher in the infected young (6.2% and 1.4%, respectively) and old groups (6.1% and 12.9%, respectively) compared with the healthy young (3.1% and 6.7%, respectively; *p* < 0.05) and old groups (3.4% and 7.4%, respectively; *p* < 0.05) (Fig. 4c). In contrast, the frequencies of IFN-γ+ (6.0%) and TNF-α+ (6.2%) cells in the lung tissue of old RMs were higher than that in young RMs (3.3% and 3.3%, respectively; *p* < 0.0001), regardless of infection (Fig. 4d, Supplementary Fig. 4). These results indicate that age does not affect virus-induced cell proliferation and immune regulation but does affect the baseline immune function of lung cells in RMs. The high level of IFN-γ and TNF-α may be a reflection of inflamm-aging in elderly RMs and may have a promoting effect on lung lesions caused by infection.

### Frequency of CXCR3+ cells increased in spleen tissues and PBMCs of SARS-CoV-2-infected old RMs

**Fig. 5:**
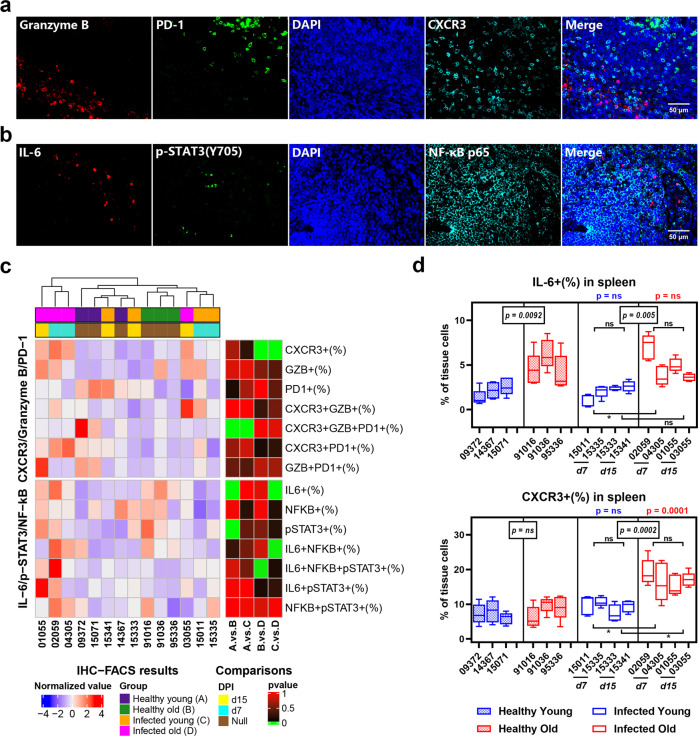
Accumulation of CXCR3+ cells in spleen tissues of old RMs infected with SARS-CoV-2. **a**, **b** mIF images showing the co-staining results of immune markers in spleen tissue sections. Red, green, and cyan indicate staining of protein markers, and blue DAPI staining indicates nucleus. **c** Staining results according to **a** and **b** of each animal are displayed in a heat map. Annotation bar indicates group and DPI of each sample; heat map on left represents the normalized average frequency of each cell subsets by R’s scale method, mapped by red-white-blue gradient squares; heat map on right represents *p* values for comparisons between each group, and square with grass green was considered to be a statistically significant difference (*p* ≤ 0.05). **d** Box plots showing the frequency of IL-6+ and CXCR3+ cells in spleen tissues in healthy young, healthy old, infected young, and infected old groups. Data calculated using five microscopy images of the spleen tissue section from each animal are shown as median with min to max. *P* value marked in blue indicates the comparison between infected and healthy young groups, and red indicates the comparison between infected and healthy old groups. Boxed *p* value represents a comparison between young and old RMs in healthy (left) or infected (right) conditions. Square brackets in the figure show a comparison between infected young and old RMs at different time points (7 and 15 DPI). *p ≤ 0.05; ns, *p* > 0.05

SARS-CoV-2 infection can cause systemic multiple organ failure and inflammation in severe cases^3,4^. However, whether systemic inflammation exists in animal models is not yet known. To solve this problem, we evaluated the characteristics of immune cells in RM spleen tissue using granzyme B, PD-1, and CXCR3 mIF staining, and IL-6, pSTAT3, and NF-κB staining (Fig. 5a, b, Supplementary Fig. 5). Compared with lung tissue, hierarchical clustering analysis of samples showed that the spleen was not strongly affected by age or infection. The frequencies of granzyme B+, PD-1+, NF-κB+, and pSTAT3+ cells in the spleen did not show significant differences among groups (Fig. 5c). However, SARS-CoV-2 infection still induced a significant increase in the frequency of CXCR3+ cells in the spleen tissue of old RMs (17.0%) compared to healthy controls (8.4%, *p* = 0.0001). Consistent with the lung tissue results, the frequency of CXCR3+ splenocytes in old RMs was higher than that in young RMs at 7 and 15 DPI. Unlike the lung, however, the frequency of IL-6+ cells in the spleen was only affected by age, with no infection-inducing effects observed. Regardless of SARS-CoV-2 infection, the frequency of IL-6+ cells in the spleen of old RMs (4.9%) was higher than that in young RMs (2.0%; *p* = 0.0002) (Fig. 5d, Supplementary Fig. 6).

**Fig. 6:**
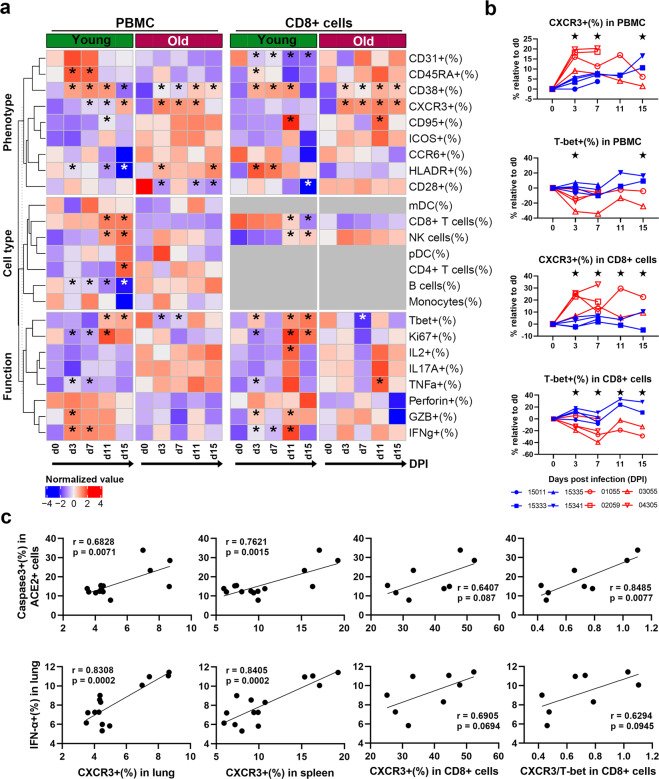
Systemic increase in CXCR3+ cells of SARS-CoV-2-infected old RMs is associated with the inflammatory microenvironment. **a** Heat map showing changes in frequency of immune cell type-, phenotype-, and function-related cell subsets in PBMCs and peripheral blood CD8+ cells from young (*n* = 4) and old RMs (*n* = 4) at 15 days after SARS-CoV-2 infection. An annotation bar indicates the age group of each sample. The average frequency was mapped by a red-white-blue gradient square. *p ≤ 0.05 compared with d0. **b** Changes in frequency of CXCR3+ or T-bet+ subsets in PBMCs and CD8+ T cells relative to d0 after infection in young and old RMs, shown as line graphs. *p ≤ 0.05 for comparison between young and old RMs at a time point. **c**, **d** Correlations between levels of CXCR3+ or T-bet+ cells in lungs, spleens, PBMCs, and CD8+ T cells and levels of IFN-α+ (**c**) and IL-6+ (**d**) cells in lung tissue are shown as scatter diagrams with linear regression curves. Dots represent each animal; *r* Pearson correlation coefficient, *p* Pearson correlation significance

We next re-examined FACS data of animal models from the previous study^15^. Our results indicated that CD8+ cells are the most infiltrated cell population in lung tissue induced by SARS-CoV-2 infection. Therefore, based on the levels of PBMCs and CD8+ cells, we evaluated differences between young and old RMs during the first 15 days of infection in terms of cell type, phenotype, and function. In addition to CD38+ and CXCR3+ cells, the frequencies of CD8+ T, natural killer (NK), and B cells, and various functional populations, such as Ki-67+, TNF-α+, granzyme B+, and IFN-γ+ cells, in PBMCs and CD8+ cells did not show significant differences in old RMs after infection but showed a SARS-CoV-2-induced effect in young RMs (Fig. 6a, Supplementary Fig. 7). These results suggest that aging affects the immune mobilization ability of RMs infected with SARS-CoV-2. However, the CXCR3+population in both PBMCs and CD8+ cells in old RMs increased rapidly compared with that in young RMs after infection, while changes in the T-bet+ population showed the opposite result (Fig. 6b, Supplementary Figs. 8–11).

### CXCR3+/T-bet+ ratio in CD8+ cells may reflect the severity of lung inflammation caused by SARS-CoV-2 infection

The young and old RMs provided excellent models to explore the relationships of the immunopathologies of SARS-CoV-2 infection in the lung, spleen, and peripheral blood. Analysis showed significant positive correlations at the cell frequency level among CXCR3+, IFN-α+, and IL-6+ cells and among NF-ΚB+ ACE2+, LC3B+ ACE2+, and caspase-3+ ACE2+ cells in lungs. These findings may indicate a series of interrelated immunopathological changes induced by a specific microenvironment in the lungs. In addition, in lung tissue, the density of ACE2+ cells was significantly negatively correlated with the frequency of CD8+, CD163+, IFN-α+, and IL-6+ cells. Moreover, the frequencies of CXCR3+ cells in the lung and spleen tissues were also significantly negatively correlated with ACE2+ cell density in the lungs. These results indicate that ACE2+ cells and the surrounding inflammatory microenvironment together influence the sensitivity to SARS-CoV-2 of RMs (Supplementary Fig. 12).

It is worth noting that the frequency of CXCR3+ cells in the lungs, spleens, PBMCs, and CD8+ cells of old RMs all increased after SARS-CoV-2 infection and showed significant positive correlations (Supplementary Fig. 12). Furthermore, the frequencies of IFN-α+ and caspase-3+ ACE2+ cells in the lung were significantly positively correlated with the frequency of CXCR3+ cells in the lung (*r* = 0.8308, *r* = 0.6828, respectively; *p* < 0.01) and spleen (*r* = 0.8405, *r* = 0.7621, respectively; *p* < 0.01), and weakly positively correlated with the frequency of CXCR3+ cells in peripheral CD8+ cells (*r* = 0.6905, *r* = 0.6407, respectively; *p* < 0.1). These findings suggested that CXCR3 could possibly be used as a marker for predicting COVID-19 severity. However, further analysis found that the CXCR3+/T-bet+ ratio in CD8+ cells was significantly positively correlated with the frequency of ACE2+ (*r* = 0.7534, *p* = 0.03), TNF-α+ (*r* = 0.7677, *p* = 0.0261), and caspase-3+ ACE2+ cells (*r* = 0.8485, *p* = 0.0077) and weakly positively correlated with the frequency of IFN-α+ cells in the lungs (*r* = 0.6294, *p* = 0.0945) (Fig. 6c).

## Discussion

Our previous study showed that, in comparison with young RMs, old RMs infected with SARS-CoV-2 exhibit more severe inflammatory infiltration and higher levels of inflammatory factors in their lungs^15^. Here, to explore the underlying mechanisms, we introduced healthy RM controls to compare the immunopathological characteristics of SARS-CoV-2 infection in young and old RMs. In addition, we introduced mIF staining based on tyramide signal amplification (TSA), which is characterized by bright signals and low background and is beneficial for improving the quality of staining and accuracy of quantitative results^23^. Based on the above, we could more accurately evaluate the age-related immunopathological mechanisms of SARS-CoV-2 infection^24^. Owing to the limitation of IHC antibodies applicable to RM tissues, we can only provide staining data for some important proteins, including inflammatory factors (IFN-α, IFN-γ, IL-6, IL-1β, and TNF-α), inflammatory cell markers (CD8, CD163, and CD11b), inflammation or interferon signaling molecules (MX1, SOCS3, pSTAT3, and NF-κB), immune function molecules (CXCR3, Granzyme B, PD-1), and other molecules (ACE2, Ki-67, active caspase-3, and LC3B).

SARS-CoV-2 is transmitted through respiratory droplets, mucosal surface contact, and a possible fecal-oral route, with ACE2 used as its binding receptor for infection^25,26^. SARS-CoV-2 infection may cause damage to epithelial and/or endothelial cells in lungs expressing ACE2, which, in turn, can cause vascular leakage, downregulation of ACE2 expression, and inflammation^27,28^. There is evidence that the expression of ACE2 in the lung is greatly reduced with aging^29^. However, compared with younger patients, elderly patients exhibit a higher severity of lung injury and higher mortality rate from COVID-19^30^. This may be owing to lower ACE2 expression in the elderly promoting activation of angiotensin II-mediated pro-inflammatory signaling pathways during infection^31,32^. Similar to that reported in COVID-19 patients, we found lower ACE2+ cell density in the lungs of healthy old RMs compared with healthy young RMs, although levels declined rapidly in both young and old RMs after infection. This suggests that direct virus infection does not fully explain the serious pathology found in old RMs.

COVID-19 pathology caused by SARS-CoV-2 infection appears to be partly based on apoptosis and autophagy of infected cells. For example, ORF6, which is located in the endoplasmic reticulum (ER) and Golgi membranes of infected cells, can induce apoptosis through caspase-3-dependent pathways and possibly through phosphorylation of c-Jun N-terminal kinase (JNK)^33^. The E protein of SARS-CoV also triggers host cell apoptosis through T-cell-mediated immune responses^34^. As a protective metabolic process, autophagy is an important mechanism for cells to resist infection. However, evidence suggests that coronaviruses, including SARS-CoV, Middle East Respiratory Syndrome (MERS)-CoV, and SARS-CoV-2, are dependent on the lysosomal proteases of the autophagy pathway to infect hosts^35,36^. Here, we found that only ACE2+ cells in old RMs showed a significant increase in apoptosis and autophagy in the early stage of infection, indicating that aging promotes SARS-CoV-2 infection at the cellular physiological level. Considering that the lesion of tissue sections we test in this study is not obvious, this phenomenon might be one of the causes of severe pathology in lung tissues of old RMs after SARS-COV-2 infection^15^.

Dysregulated inflammation is a key factor in the development of severe COVID-19, as suggested by the high expression of IL-6 in COVID-19 patients, which has a key role in inflammatory cytokine storm^37,38^. The activation of NF-κB and STAT3 by SARS-CoV-2 infection in the respiratory tract is a pre-feedback mechanism involving the IL-6-signaling pathway. In short, ADAM metallopeptidase domain 17 (ADAM17), which is activated by SARS-CoV-2 after binding to ACE2, processes the membrane form of IL-6R-α into a soluble form (sIL-6Rα). This promotes the expression of the transcription factor STAT3 in airway epithelial cells and other non-immune cells through the sIL-6Rα-IL-6 complex. The activation of STAT3 then induces the complete activation of the NF-κB pathway, leading to inflammation^39,40^. In our study, only infection in old RMs caused an increase in the proportion of IL-6-secreting cells in lung tissue, accompanied by an increase in the activation level of NF-κB in ACE2+ cells, and enhanced STAT3 and NF-κB activation in ACE2- cells. In addition, the proportion of IL-6+ cells in the spleen of old RMs was higher than that in young RMs, regardless of SARS-CoV-2 infection. This shows that aging profoundly affects COVID-19-related IL-6 signals, even in the early stage of SARS-CoV-2 infection.

The type I IFN (IFN-I) response is essential for protection against viral infections. Host-sensor recognition of pathogen-associated molecular patterns rapidly triggers the production and signal transduction of IFN-I, and induces the expression of hundreds of IFN-stimulating genes (ISGs)^41,42^. ISG-encoding proteins inhibit virus replication through a variety of mechanisms and activate various immune cells to trigger a long-term adaptive immune response against the invading virus^43,44^. However, some ISGs are involved in the regulation of cell metabolism, intracellular RNA degradation, translation arrest, and cell death, which may be harmful to the host^45^. Therefore, it is critical to understand the regulation of the IFN-I response in COVID-19 patients. We found that SARS-CoV-2 specifically induced IFN-α-secreting cells in the lungs of old RMs; however, considering that this was also accompanied by high expression of IL-6, high activation of inflammatory signals, and increased apoptosis of ACE2+ cells, it may not be conducive to virus removal.

As an important ISG-encoded protein, MX1 shows extensive antiviral activity against RNA and DNA viruses, and directly affects viral ribonucleoprotein complexes^46^. Previous transcriptome research has shown that the expression level of MX1 in COVID-19 patients is higher than that in healthy people, and increases significantly with the increase in viral load^22^. SOCS3, which is an inhibitory ISG, binds to the activation loops of receptor-related tyrosine kinases JAK2 and TYK2 through the SOCS kinase inhibitory region (KIR) to inhibit the activation of STAT, thereby attenuating the antiviral effects of IFN-I and IFN-II^47^. In vitro studies have shown that SARS-CoV infection can induce the expression of SOCS3, which may be one of the immune escape mechanisms of SARS-CoV-2^48,49^. We found that the naturally high expression of MX1+ and SOCS3+ cells in the lungs of old RMs did not increase in the early SARS-CoV-2 infection, inconsistent with the above findings. This may be because we studied the expression of MX1 and SOCS3 at the tissue cell level. However, the high level of ISG-encoding proteins constituted an abnormal immune microenvironment associated with aging, which may promote the pathological changes found in lung tissue.

SARS-CoV-2 infection and the death of lung cells trigger a local immune response, recruiting lymphocytes, macrophages, and granulocytes to respond to infection. In most cases, these recruited cells clear lung infection^50^. However, unregulated inflammatory cell infiltration can also mediate lung damage through excessive secretion of proteases and reactive oxygen species, including diffuse alveolar injury, alveolar cell desquamation, hyaline membrane formation, and pulmonary edema, leading to dyspnea and hyoxemia^51,52^. This may be another pathological basis of COVID-19. In our animal model, both old and young RMs showed increased infiltration of CD8+ cells and macrophages after infection, reflecting that SARS-CoV-2 infection caused lung lesions. We also noted that TNF-α+, IFN-γ+, and TNF-α+ IFN-γ+ cells highly infiltrate the lung tissues of old RMs regardless of infection. As TNF-α and IFN-γ work together to activate the JAK/STAT1/IRF1 axis, induce nitric oxide production, and drive caspase-8/FADD-mediated PANoptosis^53^. High expression of TNF-α and IFN-γ will aggravate SARS-CoV-2-induced inflammatory cell death in old RMs, and will eventually promote COVID-19 progress. In our previous work, we did not find differences in the protein concentration levels of TNF-α, IFN-α, IFN-γ, and IL-6 in the lung tissues between young and old RMs infected with SARS-CoV-2^15^. However, by directly observing the inflammatory cells, this study showed that the unique immune microenvironment of the old RMs still affects their pathogenesis.

The chemokine/chemokine receptor system is widely involved in the immune defense of viral infections. Chemokines recruit immune cells to the site of infection and can activate immune cells to exert direct antiviral effects^54^. Among them, the CXCL10/CXCR3 system is currently considered to be the main participant in the body’s antiviral response, especially in respiratory infections^55^. Earlier transcriptome sequencing study showed that CXCL10 gene expression is significantly upregulated in the PBMCs of COVID-19 patients, but not in bronchoalveolar lavage fluid^56^. Circulating CXCL10 concentrations in patients hospitalized in intensive care are reported to be significantly higher than that in patients with a milder clinical course^1^. Speculation of the underlying mechanism of CXCR3 in COVID-19 has mainly come from animal models. For example, a wild-type ARDS mouse model showed elevated levels of CXCL10, leading to fulminant lung inflammation, whereas CXCL10 and CXCR3 knockout mice show less-severe lung damage, indicating that CXCR3 has an important role in the progression of ARDS^57^.

In a previous study, we noticed that the CXCR3 expression of peripheral CD4+ and CD8+ T cells in old RMS increase rapidly after SARS-CoV-2 infection^15^. Here, we found for the first time that the CXCR3+ population not only increased rapidly in the lung tissue but also in the spleen, PBMCs, and CD8+ cells of old RMs infected with SARS-CoV-2, which appears to be an age-related systemic pathological feature of COVID-19. Interestingly, the frequency of T-bet+ CD8+ cells in PBMCs of old RMs decreased significantly after infection; in contrast, the proportion of CXCR3+ CD8+ cells increased. T-bet is the main transcription factor for T helper type 1 (Th1) and cytotoxic T lymphocyte (CTL) differentiation and function maintenance, thereby mediating protection against intracellular pathogens^58^. In addition, T-bet forms a migration program in effector T cells to directly activate CXCR3 to ensure proper homing to the site of inflammation^59^. Only the increased expression of CXCR3 but the insufficient expression of T-bet may not be conducive to the normal antiviral effects of CD8+ cells, which may be a cause of severe lung lesions in old RMs. However, since there is no a suitable T-bet antibody suitable for staining of paraffin tissue sections of RMs, we could not further confirm this conclusion in lung tissue.

In conclusion, our results showed that the age-related immune microenvironment in the lungs, spleens, PBMCs, and CD8+ cells significantly affected the immunopathology of SARS-CoV-2 infection, whereas the increase in inflammatory factor-secreting cells in the lungs and systemic accumulation of CXCR3+ cells are associated with necrosis and inflammatory activation of ACE2+ lung cells. Owing to lack of commercial antibodies suitable for RMs, we can only detect a small number of immunological markers on tissue levels. Although these results provide support for the immunopathological mechanism of COVID-19, further functional verification is still needed.

## Materials and methods

### Animal experiments and ethics statement

Healthy young (male, *n* = 7, 5–11 years old) and old (male, *n* = 7, 16–29 years old) RMs without simian type D retrovirus (SRV), simian T lymphotropic virus (STLV), ceropithecine herpesvirus 1 (BV) and coronavirus (CoV) were sourced from the Laboratory Animal Center, Kunming Institute of Zoology (KIZ), Chinese Academy of Sciences (CAS). Among them, four young (#15011, #15333, #15335, and #15341) and four old (#01055, #02059, #03055, and #04305) RMs were intratracheally inoculated with 1 × 10^7^ TCID50 SARS-CoV-2 in a 2 mL volume by bronchoscope, whereas three young (#09372, #14367, and #15071) and three old (#91016, #91036 and #95336) RMs were served as healthy controls without infection. The SARS-CoV-2 strain (NMDC number: NMDCN0000HUI) was obtained from the Guangdong Provincial CDC, Guangdong, China. In order to obtain lung and spleen tissues, half of the animals were euthanized on 7 DPI and half on 15 DPI, whereas animals in the healthy group were killed without exposure to SARS-CoV-2. PBMC samples of the infected group were collected at 0, 3, 7, 11, and 15 DPI. All animal experiments were performed in the Kunming National High-Level Biosafety Research Center for Non-Human Primates, Center for Biosafety Mega-Science, KIZ, CAS, and according to the guidelines approved by the Ethics Committee of the Kunming Institute of Zoology (approval no.: IACUC20005) in accordance with the suggestion of “The use of non-human primates in research” (Weatherall D, 2006)”.

### IHC and mIF staining

We used IHC staining to detect SARS-CoV-2 in lung tissues, and mIF staining based on the TSA method to detect protein markers in lung and spleen tissues^23^. In brief, the formalin-fixed paraffin-embedded (FFPE) tissue sections after deparaffinization and hydration treatment were repaired in a pressure cooker with Tris-EDTA (pH = 8.0) solution for 2 m, and then cooled to room temperature. After the endogenous peroxidase inactivation and serum blocking steps, the sections were incubated with the primary antibody for 2 h at room temperature, and then incubated with the horseradish peroxidase-labeled secondary antibody for 1 h after being washed with Tris-HCl (pH = 7.4) buffer. In the IHC staining steps, the sections were then treated with DAB staining kit (Solarbio, China) to make the target protein appear brown, and then treated with Mayer’ Hematoxylin solution (Solarbio, China) to make the nucleus appear blue. After labeling, the sections were mounted and photographed with an Olympus BX46 microscope. In the mIF-staining steps, the sections were treated with a borate buffer (pH = 8.5) containing H_2_O_2_ (0.003%), 4-Iodophenol (50–500 μg/mL), Dextran sulfate sodium (2%), and fluorescein-labeled tyramine (AAT Bioquest, USA) (5–20 mg/mL) for 10–20 m at room temperature. Then immediately place the slices in citrate buffer (pH = 6.0) and microwave for 10 m to remove the bound antibody. After returning to room temperature, the slices are subjected to the next two rounds of staining cycles and finally stained with iFluor488, Cy3, and Cy5. After the fluorescent labeling, the sections were processed with Sudan Black B (0.5%) and 4′,6-diamidino-2-phenylindole (5 μg/mL), then mounted and photographed with a Leica DMI4000 fluorescence microscope. The detailed information of all antibodies involved was shown in Supplementary Table S1.

### Quantitative analysis of microscopic images

For IHC microscopic image, we first used plug-in IHC Profiler of ImageJ (V1.53, NIH) software to disassemble the colors of hematoxylin and DAB into two channels of eight-bit images. For the mIF microscopic image, we disassembled it into four channels of eight-bit images representing the signal of the nucleus and three protein markers. Subsequently, we used the Dns Macro Example (De Novo Software, USA) plug-in of ImageJ to analyze the eight-bit images of each channel, calculate the pixel area and position of each cell based on nucleus signal, as well as the signal characteristics of each marker on each cell, and finally export these values as a csv file in matrix format. Then, we imported the csv file into R (V4.0.3, GNU project) software, clustered the cells with positive and negative signals of each marker through the Iterative Self-Organizing Data Analysis Technique (ISODATA) and the k-means clustering algorithms, and further calculated the relative signal value of each marker on each cell. Finally, we output all the information of each marker of all cells as an FCS file and sent it to Flowjo (V10.5.3, BD Biosciences) software for quantitative analysis (IHC-FACS analysis). The detailed analysis process was shown in Supplementary Fig. S13, S14.

### Flow cytometry

For the study on the immune characteristics of peripheral blood in animal models, we adopted a flow cytometry strategy using four antibody panels, which analyze PBMC and CD8+ cells according to three sections: cell type, phenotype, and function. Detailed antibody panels for flow cytometry are provided in Supplementary Table S2. In brief, PBMCs were thawed from liquid nitrogen and then incubated with antibodies to surface antigens. The surface-labeled cells were then fixed and permeabilized using a transcription factor staining buffer set (eBioscience, USA) for Ki-67 and T-bet staining. For analysis of immune cell function, the PBMCs were resuspended in Roswell Park Memorial Institute (RPMI)-1640 medium with 10% fetal bovine serum, PMA (50 ng/mL), ionomycin (1 μmol/L), brefeldin A (5 μg/mL), and monensin (2 μmol/L), then incubated at 37°C for 5 h in a cell incubator. After surface staining and treatment using a Cytofix/Cytoperm Kit (BD Biosciences, USA), intracellular staining steps for IFN-γ, TNF-α, granzyme B, and other intracellular proteins were performed. The acquisition of at least one million cells was performed using a FACSCelesta flow cytometer (BD Biosciences, USA), and data analysis was performed using FlowJo software.

### Statistical analysis

Each staining panel of tissue sections of the left upper lung, right upper lung, and spleen from each animal was taken with five microscopic images at 200 times magnification (627 × 427 μm) to calculate the frequency of cells expressing protein markers. Based on these data, we then used Tukey’s multiple comparisons test post nested one-way analysis of variance (ANOVA) in GraphPad Prism software (v8, GraphPad) to compare the differences in the cell frequency among each group. For flow cytometry data, we used the Fisher’s LSD test post two-way ANOVA in GraphPad Prism software to compare the difference of each test index between a time point after infection and 0 DPI, or the difference between young and old RMs at a certain time point. The correlation coefficient (*r*) and *p* value were calculated by Pearson’s method in R. For all comparison results, *p* ≤ 0.05 is considered statistically significant. The data in the text content were displayed as mean values.

## Supplementary information

Supplementary materials

## Data Availability

All data that support the findings of this study are available from the corresponding author upon reasonable request.
