# Supplementary material for: Pro-inflammatory microenvironment and systemic accumulation of CXCR3+ cell exacerbate lung pathology of old rhesus macaques infected with SARS-CoV-2
Source: Signal Transduct Target Ther. 2021 Sep 1;6:328. doi: 10.1038/s41392-021-00734-w (PMC8409077; doi:10.1038/s41392-021-00734-w)
Supplement: Supplementary file 1 — Supplementary materials [file 41392_2021_734_MOESM1_ESM.pdf]

Supplementary Materials for

Pro-inflammatory microenvironment and systemic accumulation of CXCR3+ cell exacerbate lung pathology of old rhesus macaques infected with SARS-CoV-2

Hong-Yi Zheng, Xiao-Yan He, Wei Li, Tian-Zhang Song, Jian-Bao Han, Xiang Yang, Feng-Liang Liu, Rong-Hua Luo, Ren-Rong Tian, Xiao-Li Feng, Yu-Hua Ma, Chao Liu, Ming-Hua Li, Yong-Tang Zheng

Correspondence to: zhengyt@mail.kiz.ac.cn

This PDF file includes:

Figures. S1 to S14
Tables S1 to S2

Table S1 Antibodies for immunohistochemistry and immunofluorescence

Primary antibodies

Antibody	Company	Catalog	Host species
SARS-COV-2 Nucleoprotein	Sino biological	40143-R019	rabbit
CD8α	CST	85336	rabbit
CD163	Abcam	ab182422	rabbit
CD11b	Abcam	ab133357	rabbit
ACE2	Service bio	GB11267	rabbit
STAT3 (phospho Y705)	Abcam	ab76315	rabbit
NF-KB (P65)	Santa Cruz	sc-514451	mouse
Cleaved Caspase-3 (Asp175)	CST	9664	rabbit
LC3B	Abcam	ab63817	rabbit
MX1	Proteintech	13750-1-AP	rabbit
SOCS3	ABclonal	A0694	rabbit
PD-1	Abcam	ab137132	rabbit
Granzyme B	Abcam	ab255598	rabbit
CXCR3	BD bioscience	557183	mouse
TNF-α	Santa Cruz	sc-52746	mouse
IFN-γ	Abcam	ab218426	rabbit
Ki67	Abcam	ab15580	rabbit
IFN-α	Santa Cruz	sc-373757	mouse
IL-6	Sino biological	90197-RP02	rabbit
IL-1β	Service bio	GB12113	mouse

Second antibodies

Antibody	Company	Catalog	Host species
Goat Anti-Mouse IgG H&L (HRP)	Abcam	ab7068	goat
Goat Anti-Rabbit IgG H&L (HRP)	Abcam	ab7090	goat
Goat Anti-Mouse IgG Polymer -HRP	Boster	SV0001	goat
Goat Anti-Mouse IgG Polymer -HRP	Boster	SV0002	goat

Table S2 Antibodies for flow cytometry

Panel: Immune cell type			
**Antibody**	**Fluorochrome**	**Clone**	**Source**
CD123	BV421	7G3	BD Pharmigen
CD20	BV510	2H7	BD Pharmigen
CD45	BV605	D058-1283	BD Pharmigen
CD56	BV650	NCAM-16.2	BD Pharmigen
CD14	BV786	M5E2	BD Pharmigen
CD11C	FITC	3.9	BD Pharmigen
NKG2A	PE	REA110	Miltenyi Biotec
CD16	PE-CF594	3G8	BD Pharmigen
CD4	PerCP-Cy5.5	L200	BD Pharmigen
HLA-DR	APC	G46-6	BD Pharmigen
CD8	APC-R700	RPA-T8	BD Pharmigen
CD3	APC-Cy7	SP34-2	BD Pharmigen

Panel: Immune activation			
**Antibody**	**Fluorochrome**	**Clone**	**Source**
CD28	BV510	CD28.2	BD Pharmigen
CXCR3	BV605	G025H7	Biolegend
CD95	BV786	DX2	Biolegend
HLA-DR	FITC	G46-6	BD Pharmigen
ICOS	PE-Dazzle594	C398.4A	Biolegend
CCR6	PerCP-Cy5.5	G034E3	Biolegend
CD38	APC	AT-1	NIH
CD8	APC-R700	RPA-T8	Biolegend
CD3	APC-Cy7	SP34-2	Biolegend

Table S2 Antibodies for flow cytometry (continued table)

Panel: Immune regulation

**Antibody**	**Fluorochrome**	**Clone**	**Source**
CD28	BV510	CD28.2	BD Pharmigen
T-bet	BV605	4B10	Biolegend
CD95	BV786	DX2	Biolegend
Ki67	PE	B56	BD Pharmigen
CD31	PE-Dazzle594	WM59	Biolegend
CD45RA	APC	5H9	BD Pharmigen
CD8	APC-R700	RPA-T8	BD Pharmigen
CD3	APC-Cy7	SP34-2	BD Pharmigen

Panel: Immune function

**Antibody**	**Fluorochrome**	**Clone**	**Source**
Granzyme B	Pacific Blue	GB11	Biolegend
CD28	BV510	CD28.2	BD Pharmigen
IL-2	BV650	MQ1-17H12	Biolegend
CD95	BV786	DX2	Biolegend
Perforin	FITC	Pf-344	Mabtech
TNF-α	PE-Dazzle594	MAb11	Biolegend
IL-17A	PerCP-Cy5.5	BL168	Biolegend
IFN-γ	APC	4S.B3	Biolegend
CD8	APC-R700	RPA-T8	BD Pharmigen
CD3	APC-Cy7	SP34-2	BD Pharmigen

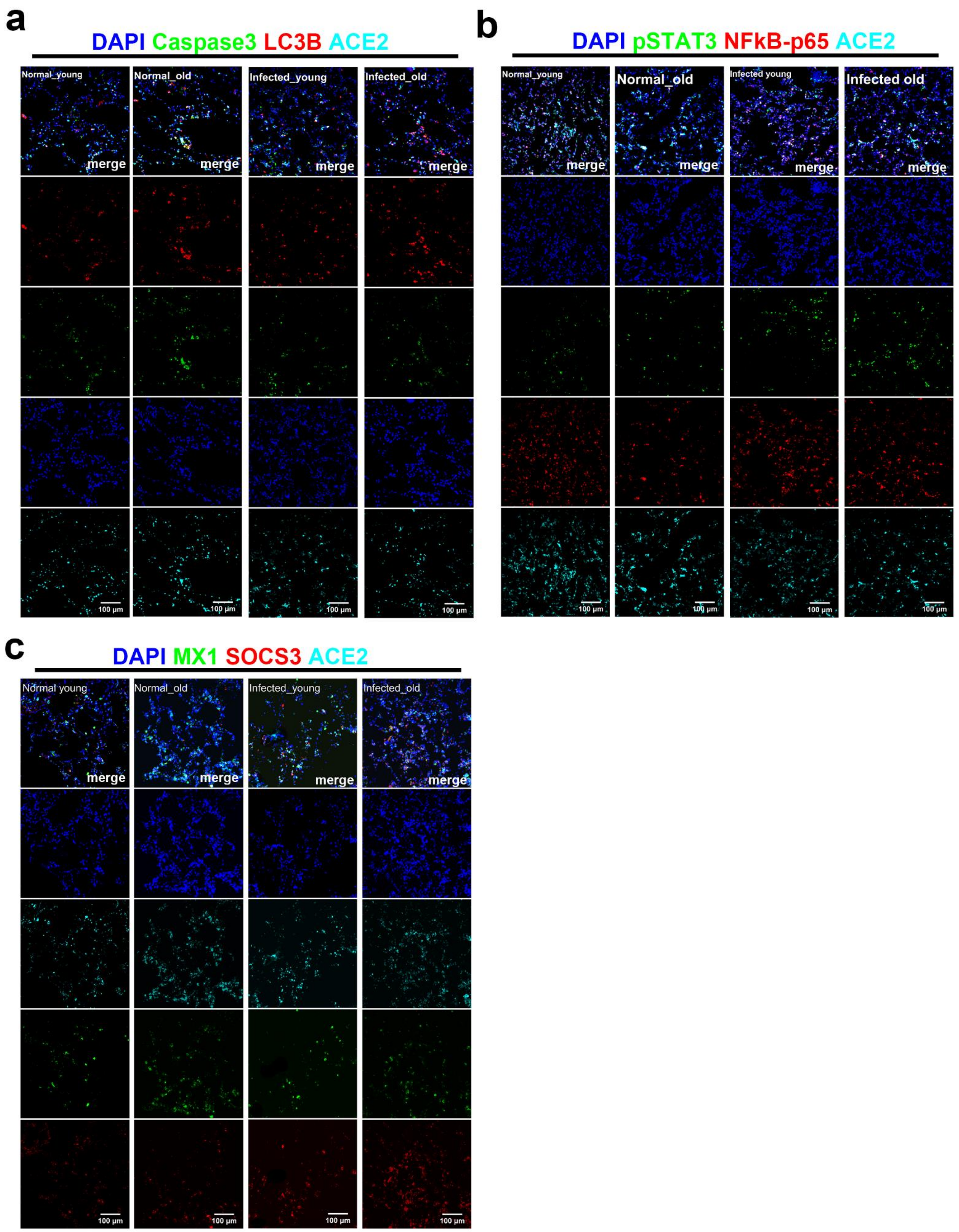

Figure S1. Representative images for mIF staining of ACE2+ and ACE2- cells in lung tissue.

mIF images showing three sets of co-staining results of ACE2 with two other proteins in lung tissue sections, representing cell death (a), inflammatory signal activation (b), and IFN-signal activation (c) of ACE2+ and ACE2− cells in normal young, normal old, infected young and infected old groups. Red, green, and cyan indicate staining of protein markers, and blue DAPI staining indicates nucleus. Each group consists of 4 channels and their merged image.

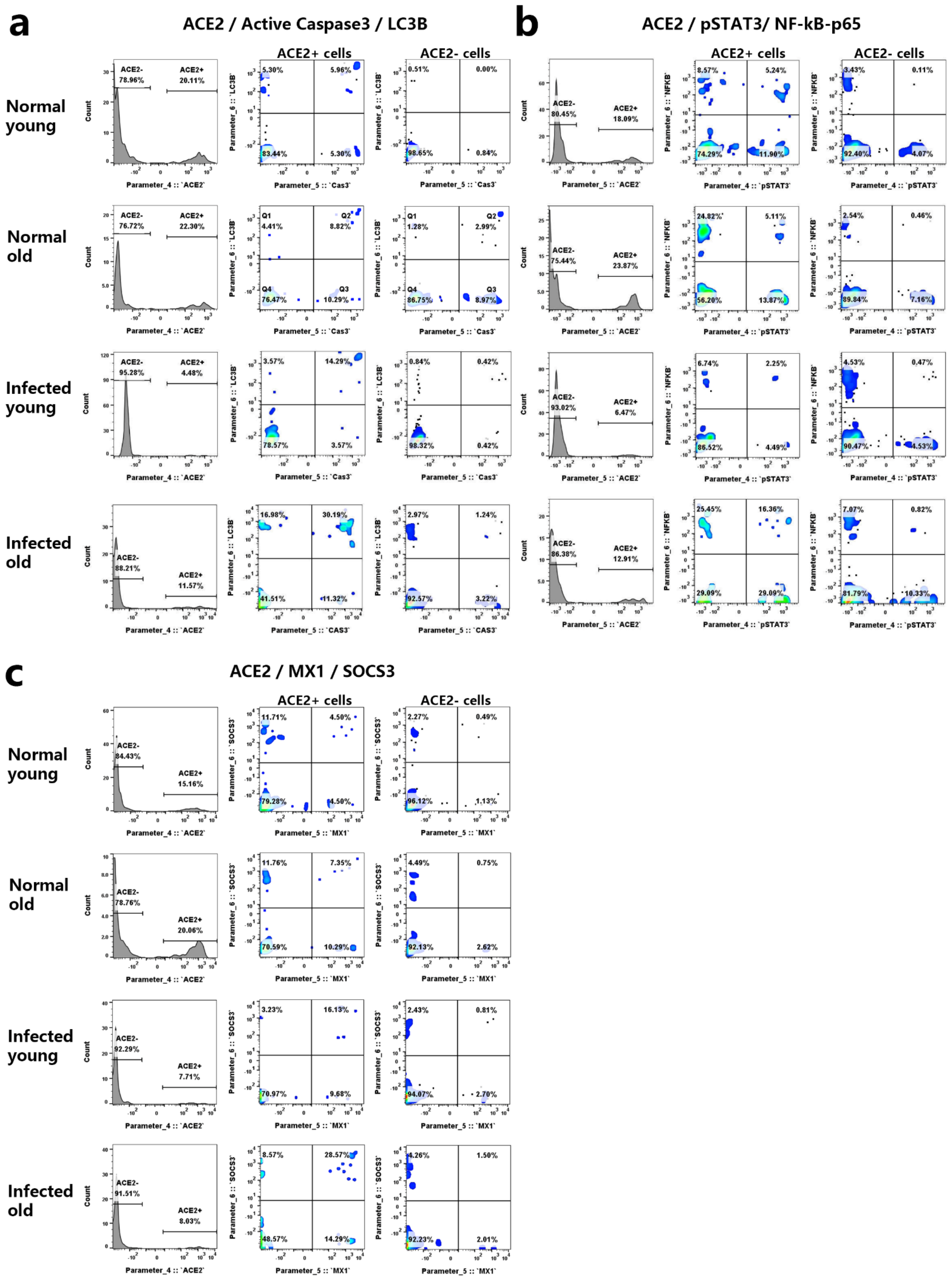

Figure S2. Representative IHC-FACS results for mIF staining of ACE2+ and ACE2- cells in lung tissue.

Flow cytometry images showing three sets of co-staining results of ACE2 with two other proteins in lung tissue sections, representing cell death (a), inflammatory signal activation (b), and IFN-signal activation (c) of ACE2+ and ACE2− cells in normal young, normal old, infected young and infected old groups.

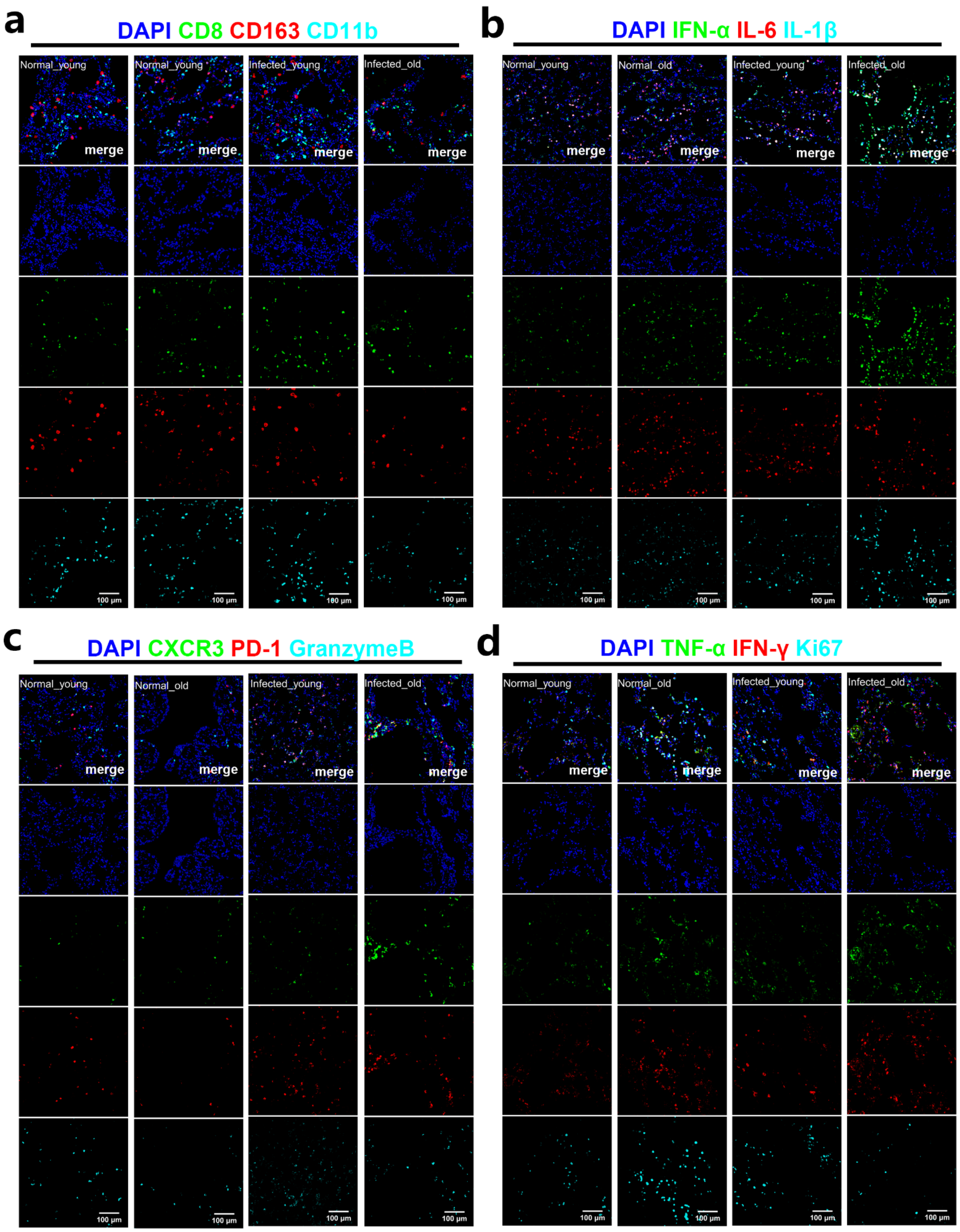

Figure S3. Representative images for mIF staining of immune molecules in lung tissue.

mIF images showing the co-staining results of inflammatory cells (a), inflammatory factors (b) and immune function related proteins (c, d) in lung tissue sections of normal young, normal old, infected young and infected old groups. Red, green, and cyan indicate staining of protein markers, and blue DAPI staining indicates nucleus. Each group consists of 4 channels and their merged image.

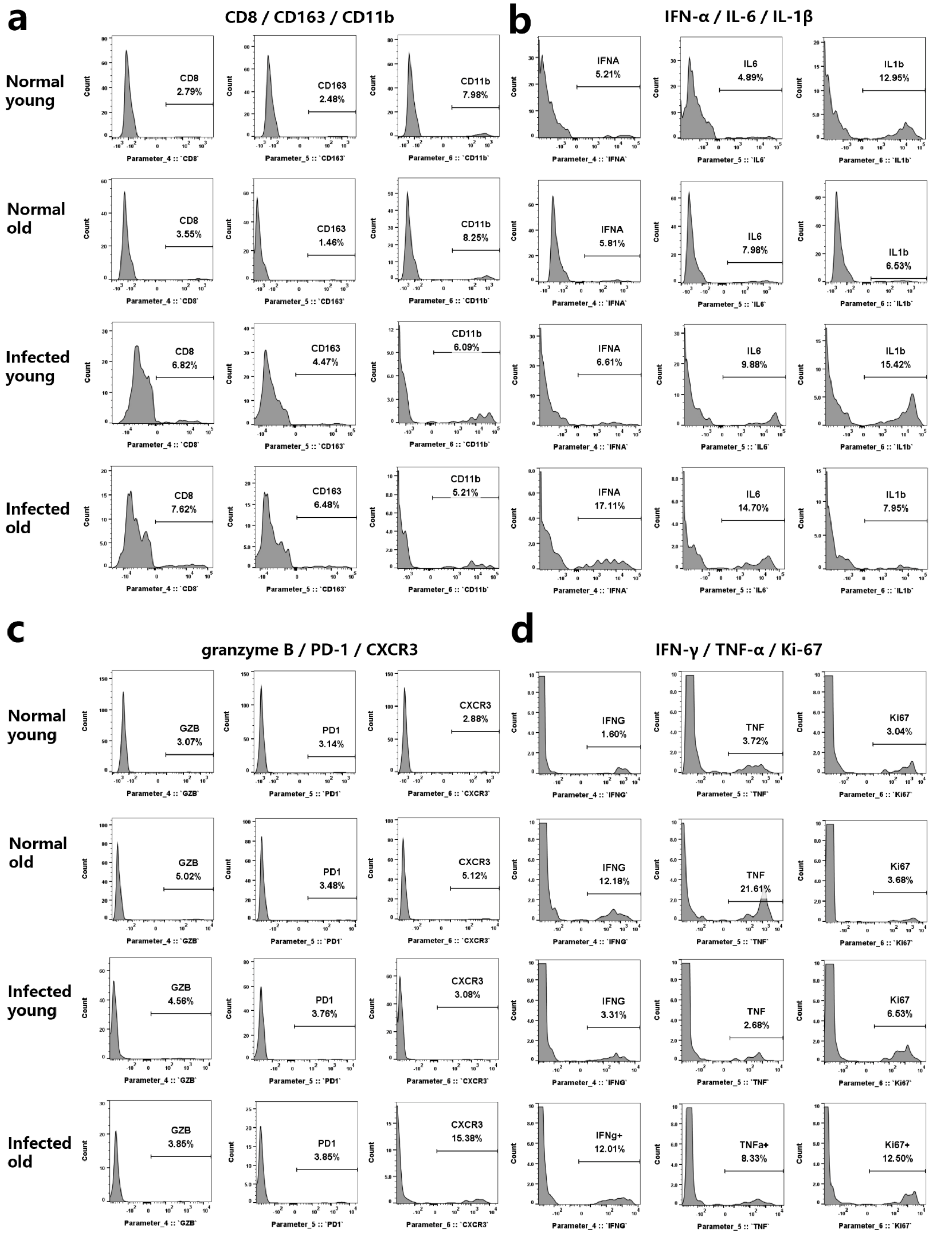

Figure S4. Representative IHC-FACS results for mIF staining of immune molecules in lung tissue.

Flow cytometry images showing three sets of co-staining results of inflammatory cells (a), inflammatory factors (b) and immune function related proteins (c, d) in lung tissue sections of normal young, normal old, infected young and infected old groups.

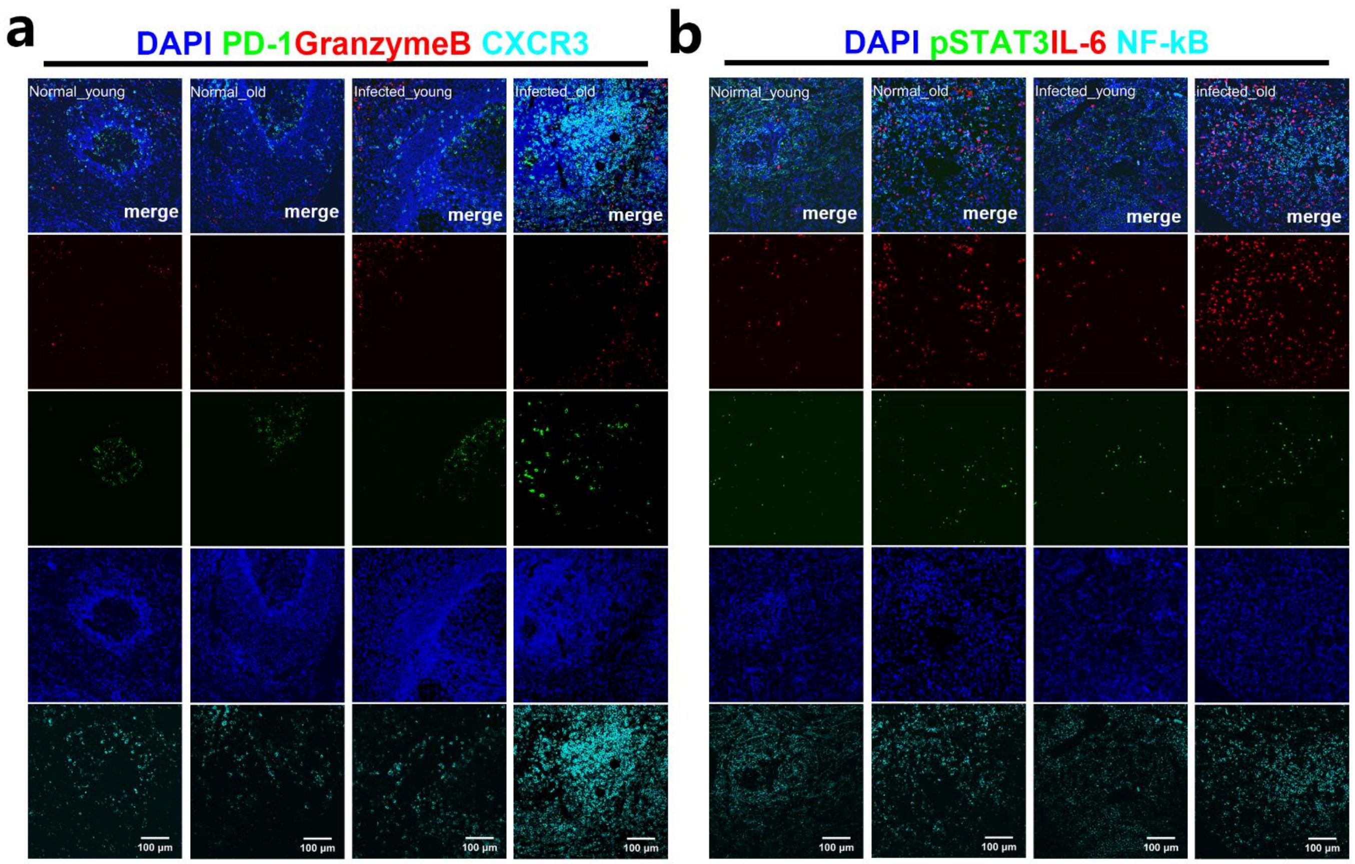

Figure S5. Representative images for mIF staining of immune molecules in spleen tissue.

mIF images showing the co-staining results of immune function immune function (a) and inflammatory signal (b) in spleen tissue sections of normal young, normal old, infected young and infected old groups. Red, green, and cyan indicate staining of protein markers, and blue DAPI staining indicates nucleus. Each group consists of 4 channels and their merged image.

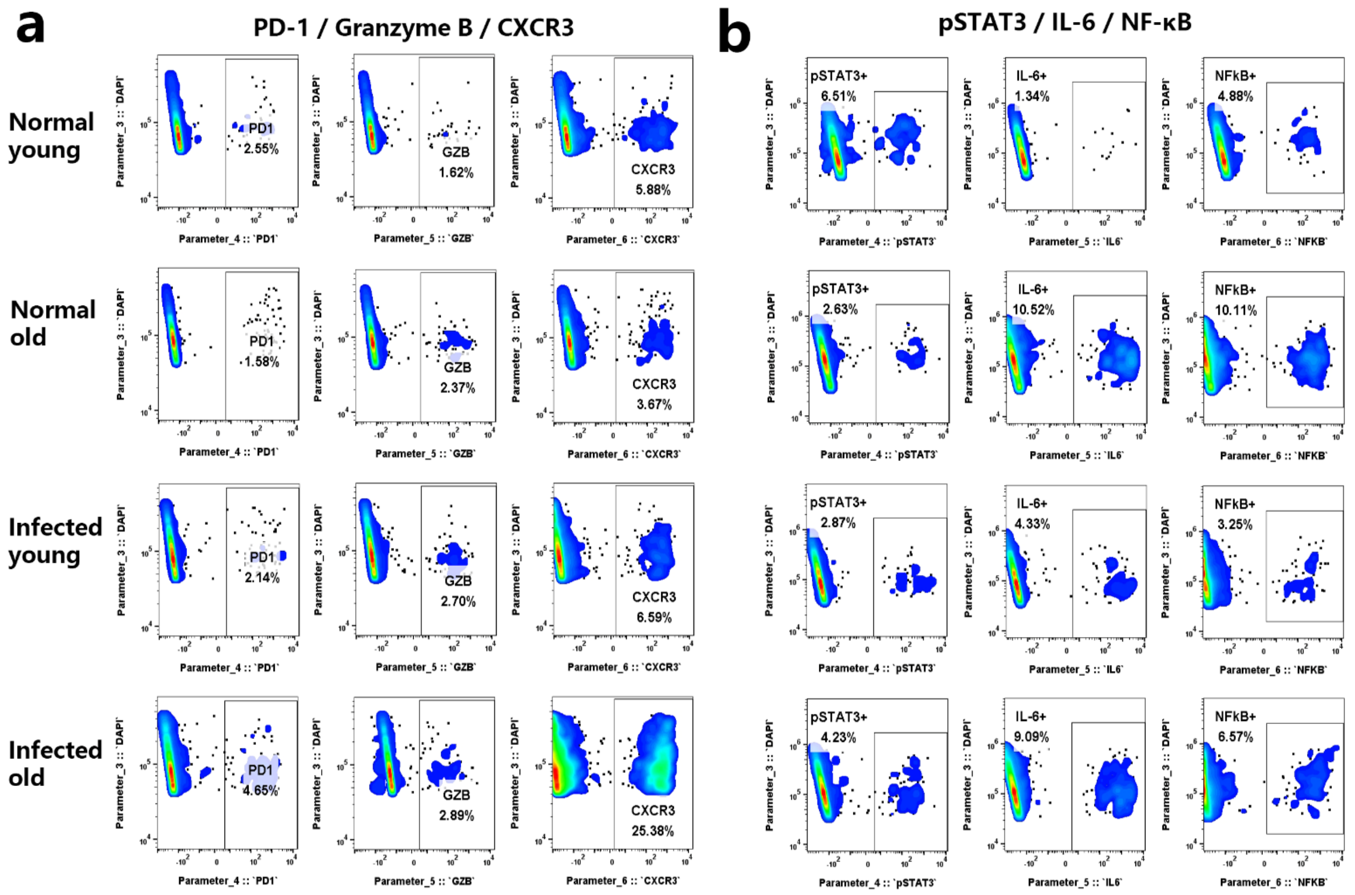

Figure S6. Representative IHC-FACS results for mIF staining of immune molecules in spleen tissue.

Flow cytometry images showing three sets of immune function immune function (a) and inflammatory signal (b) in spleen tissue sections of normal young, normal old, infected young and infected old groups.

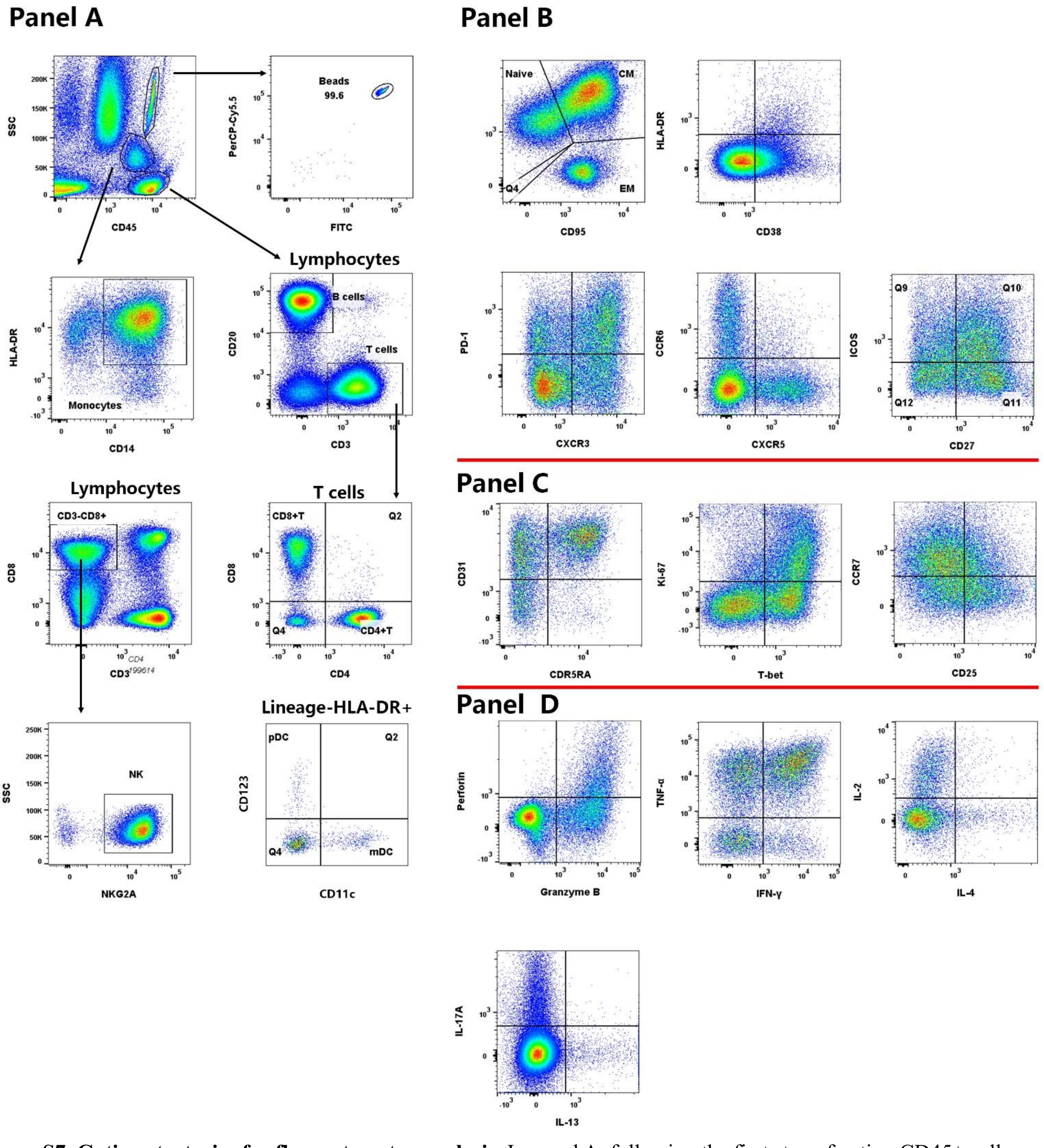

Figure S7. Gating strategies for flow cytometry analysis. In panel A**,** following the first step of gating CD45+ cells, lymphocytes were gated by FSC and SSC, CD14+HLA-DR+ were selected as monocytes, and counting beads are characterized by high intensity fluorescence. Then, B cells, T cells and NK cells were gated as CD3-CD20+, CD3+CD20-, CD3-CD8+NKG2A+, respectively. T cells were further divided into CD4+CD8- and CD4-CD8+ subsets. CD123 and CD11c can be used to divide CD3-CD20-CD14-HLA-DR+ subset into pDC and mDC. Through different antibody panels and 12-color flow cytometry, the activation (panel B), regulation (panel C) and functional characteristics (panel D) of total PBMC and CD8+ cells in rhesus models were continuously analyzed.

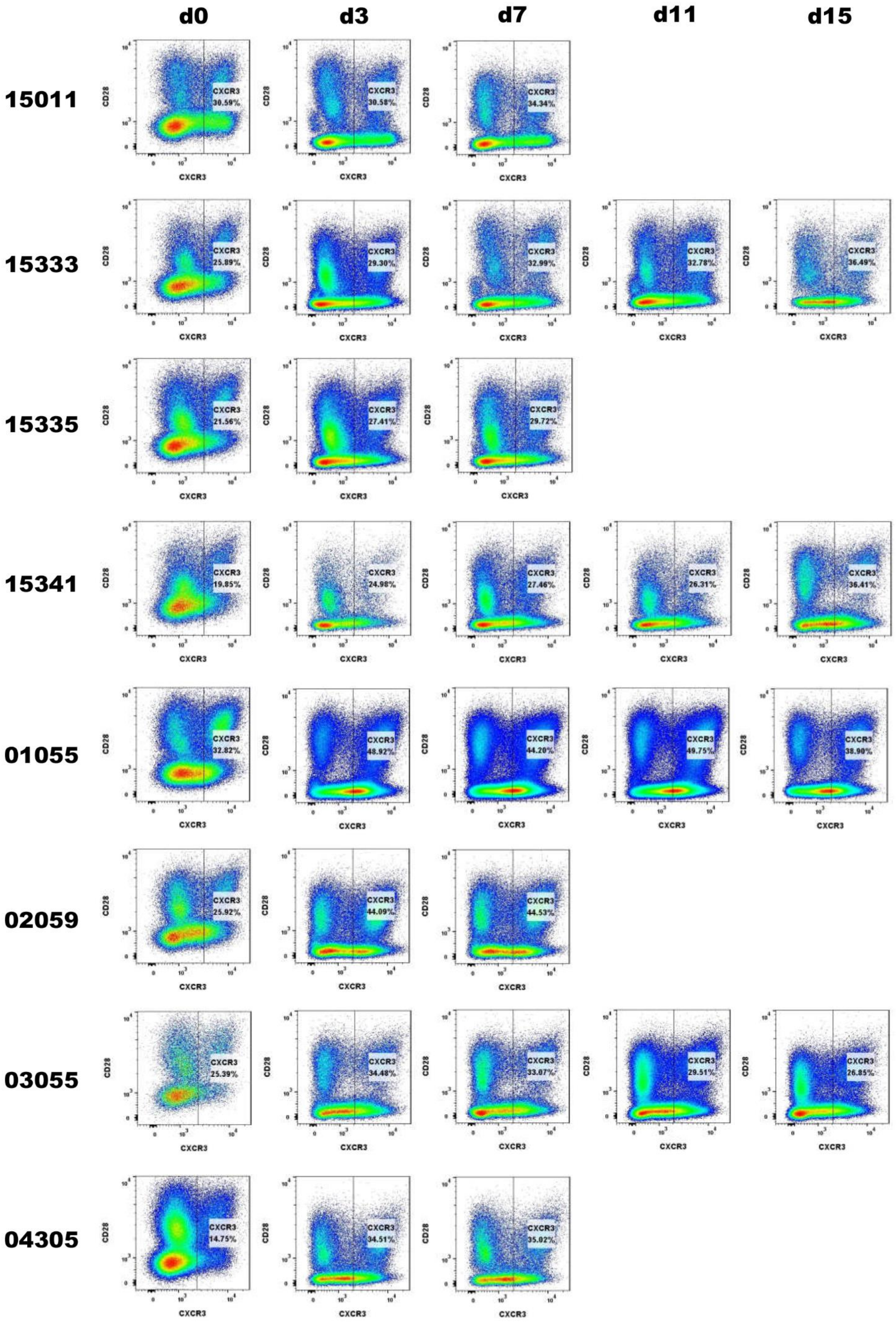

Figure S8. Flow cytometry results of the CXCR3 expression in PBMC. Pseudo-color scatter plot showing the frequency of CXCR3+ cells in PBMC during infection in each animal.

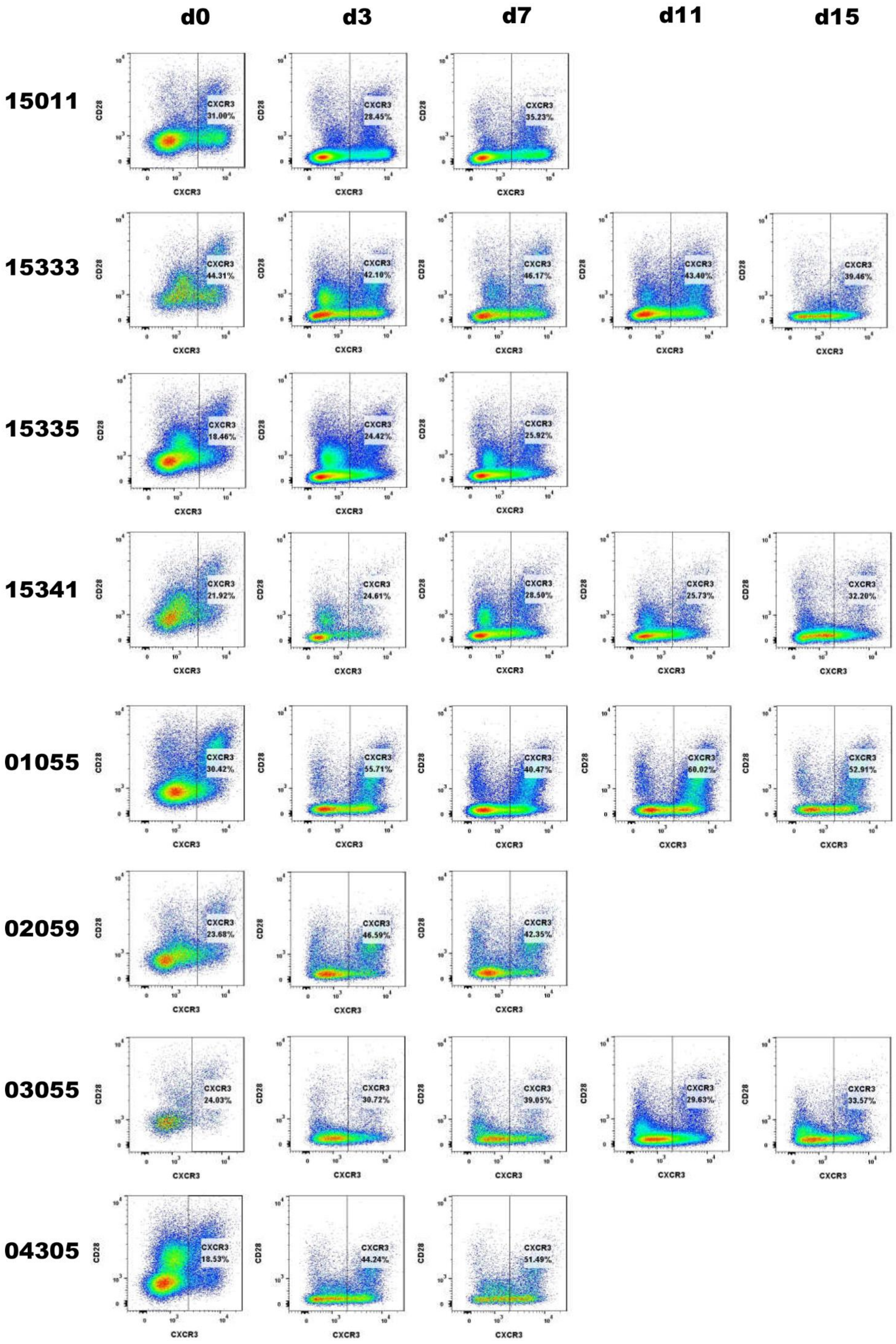

Figure S9. Flow cytometry results of the CXCR3 expression in CD8+ cells. Pseudo-color scatter plot showing the frequency of CXCR3+ cells in CD8+ cells during infection in each animal.

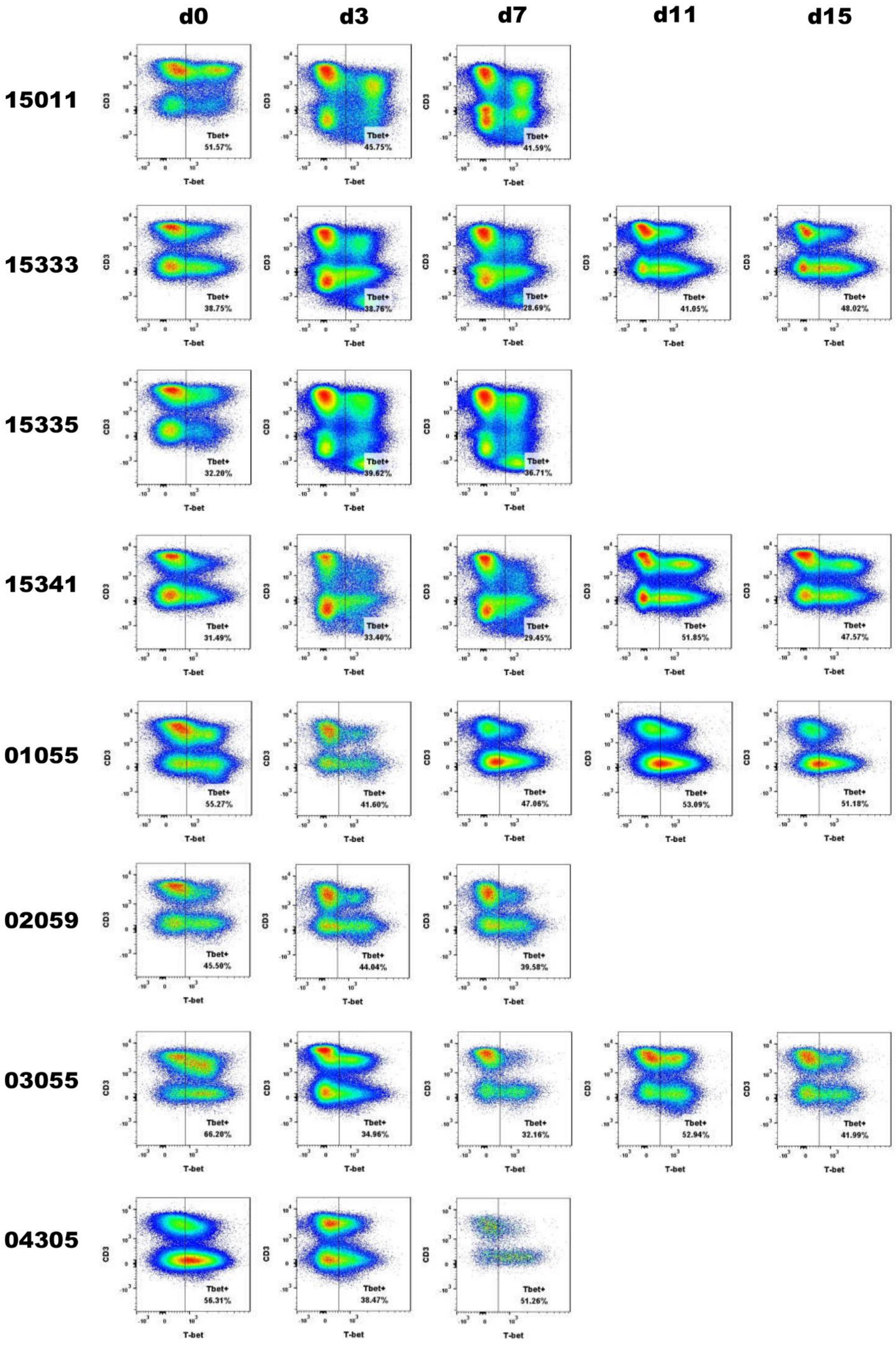

Figure S10. Flow cytometry results of the T-bet expression in PBMC. Pseudo-color scatter plot showing the frequency of CXCR3+ cells in PBMC in each animal during infection.

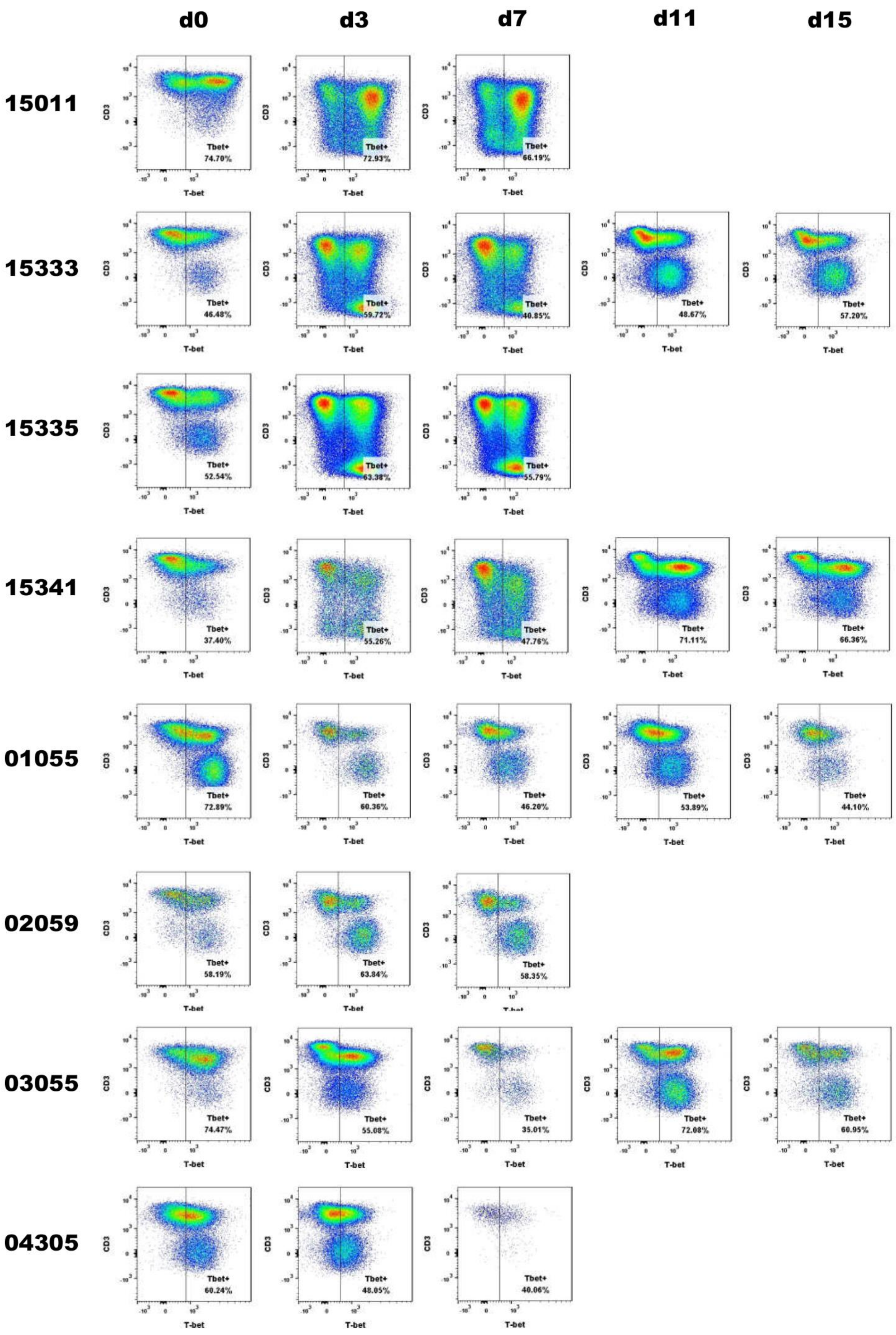

Figure S11. Flow cytometry results of the T-bet expression in CD8+ cells. Pseudo-color scatter plot showing the frequency of CXCR3+ cells in CD8+ cells in each animal during infection.

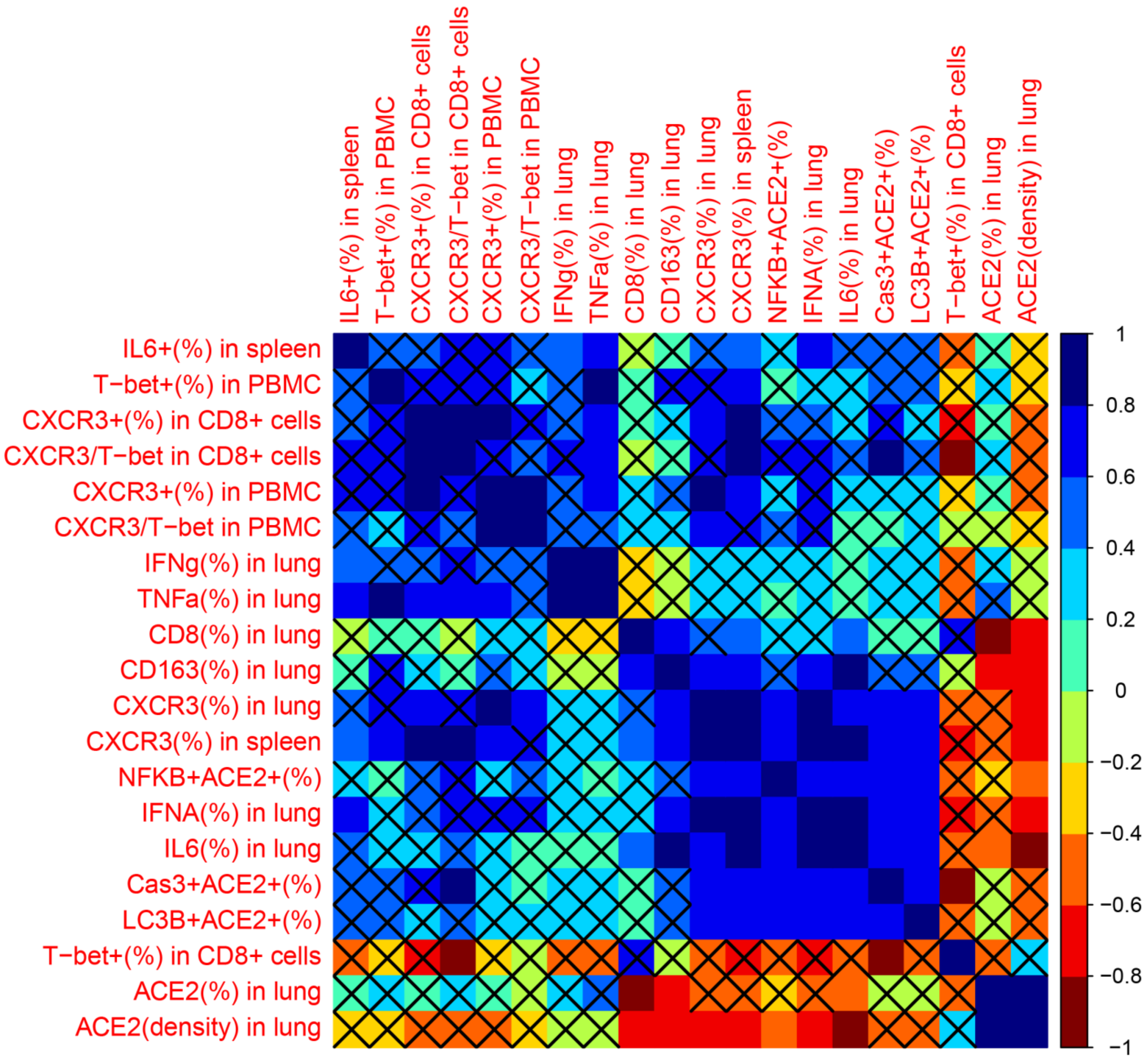

Figure S12. The correlation matrix of immunopathological indicators.

We calculated the average value of immunopathological indicators obtained from multiple microscopic images of each animal's lung and spleen tissue, as well as the average value of the immune indicators of PBMC and CD8+ cells during the infection period of each anima, and then performed Pearson's correlation analysis on them to construct a correlation matrix. The color of the square maped the correlation coefficient, and the cross indicates that the correlation is not significant (Pearson's $p > 0.05$).

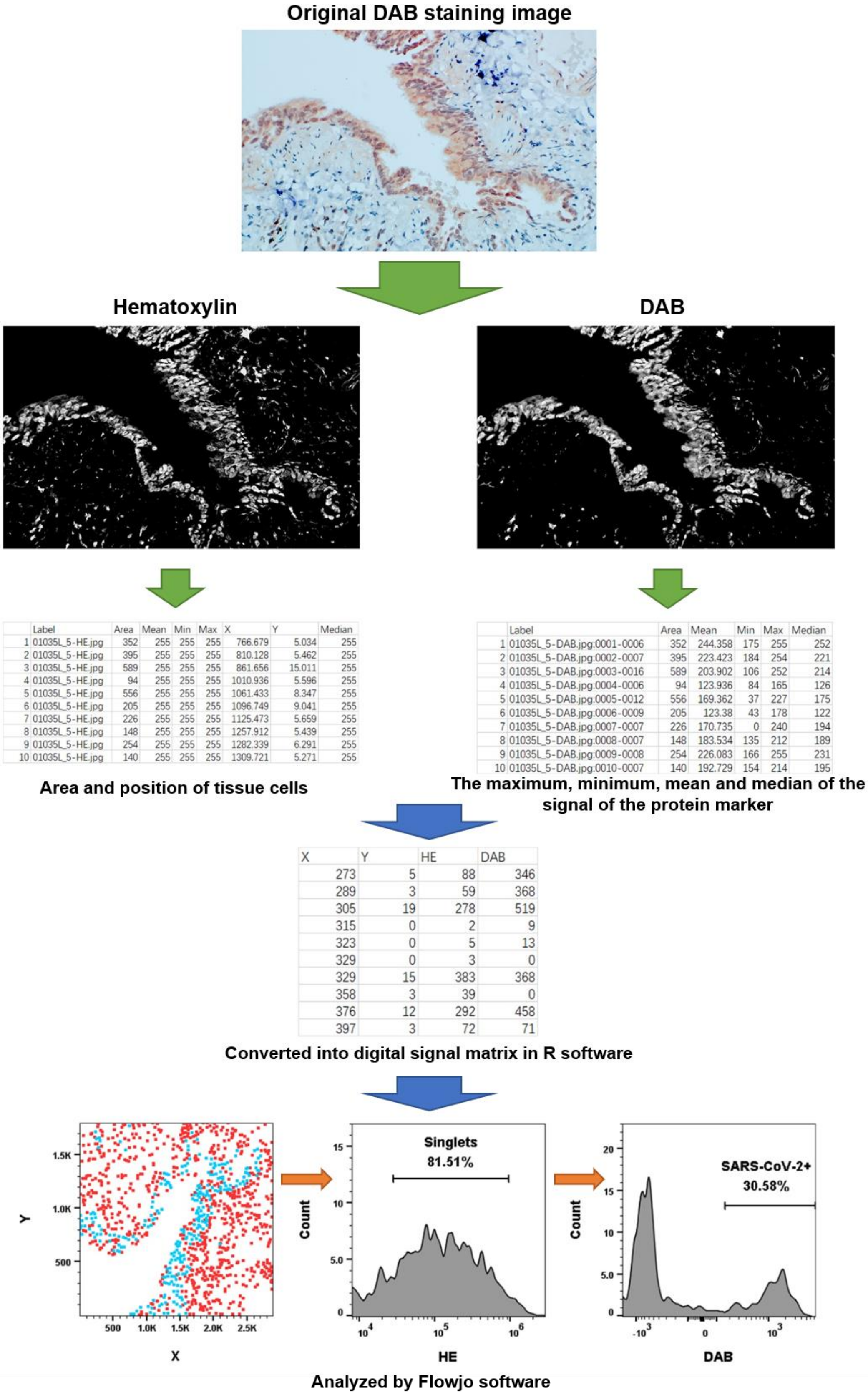

Figure S13. The process of converting DAB staining image into fcs data.

The DAB staining image was split into two 8-bit images of hematoxylin and DAB to calculate the position and area of the cells in the image, as well as the maximum, minimum, median and mean values of the DAB signal of each cell. These data were passed to the R software to determine the positive signal threshold through a clustering algorithm, calculate the relative DAB signal intensity of each cell, and export a fcs file in the form of a digital matrix. Finally, these fcs files are analyzed by Flowjo software according to the method of flow cytometry.

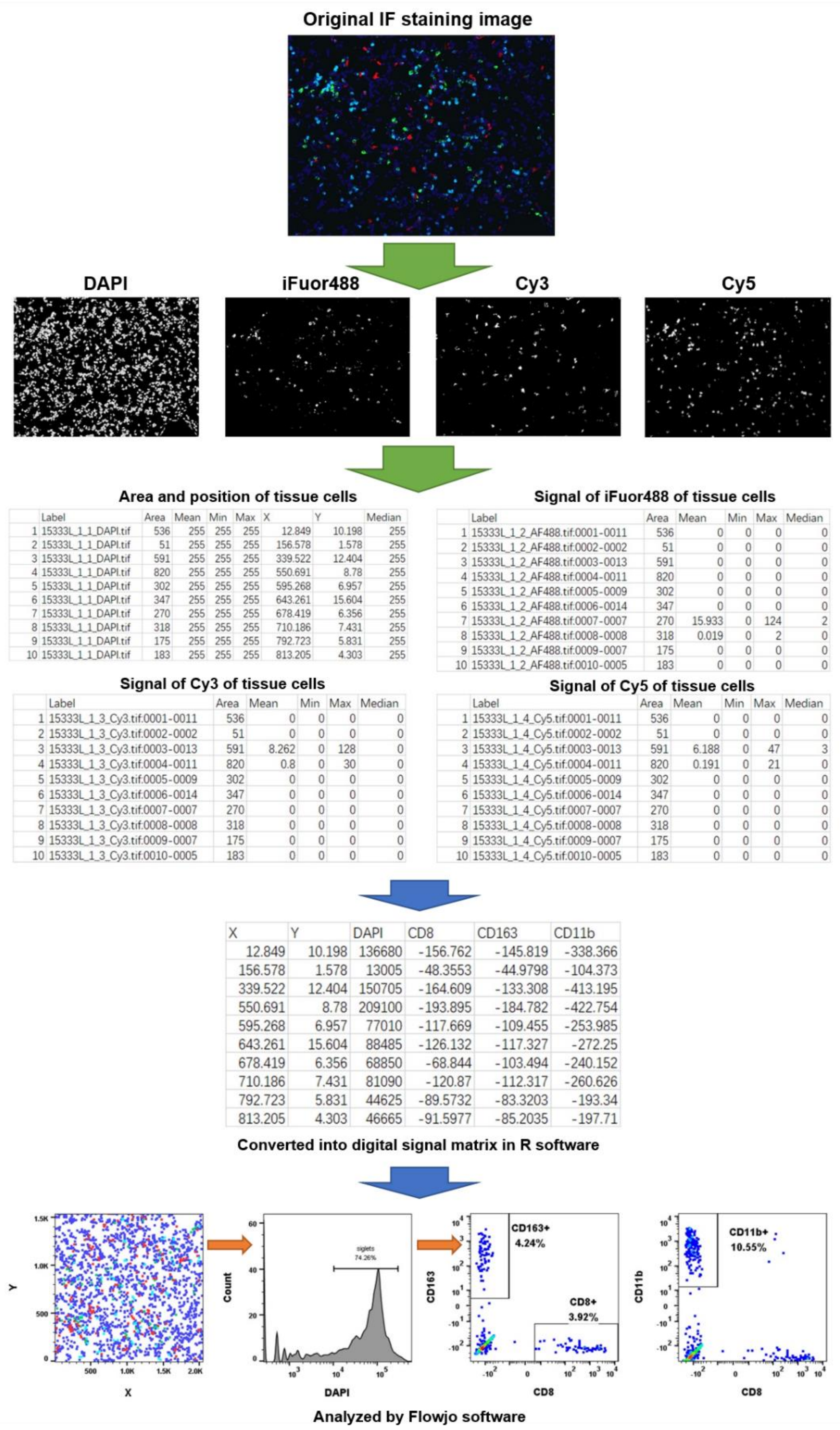

Figure S14. The process of converting mIF staining image into fcs data.

The IF staining image was split into four 8-bit images of DAPI, iFuor488, Cy3 and Cy5 to calculate the position and area of the cells in the image, as well as the maximum, minimum, median and mean values of the fluorescence signal of each cell. These data were passed to the R software to determine the positive signal threshold through a clustering algorithm, calculate the relative fluorescence signal intensity of each cell, and export a fcs file in the form of a digital matrix. Finally, these fcs files are analyzed by Flowjo software according to the method of flow cytometry.
